# Endocrine-Disrupting Chemicals: Associated Disorders and Mechanisms of Action

**DOI:** 10.1155/2012/713696

**Published:** 2012-09-06

**Authors:** Sam De Coster, Nicolas van Larebeke

**Affiliations:** Study Centre for Carcinogenesis and Primary Prevention of Cancer, Department of Radiotherapy and Experimental Cancerology, Ghent University Hospital, De Pintelaan 185 3K3, 9000 Ghent, Belgium

## Abstract

The incidence and/or prevalence of health problems associated with endocrine-disruption have increased. Many chemicals have endocrine-disrupting properties, including bisphenol A, some organochlorines, polybrominated flame retardants, perfluorinated substances, alkylphenols, phthalates, pesticides, polycyclic aromatic hydrocarbons, alkylphenols, solvents, and some household products including some cleaning products, air fresheners, hair dyes, cosmetics, and sunscreens. Even some metals were shown to have endocrine-disrupting properties. Many observations suggesting that endocrine disruptors do contribute to cancer, diabetes, obesity, the metabolic syndrome, and infertility are listed in this paper. An overview is presented of mechanisms contributing to endocrine disruption. Endocrine disruptors can act through classical nuclear receptors, but also through estrogen-related receptors, membrane-bound estrogen-receptors, and interaction with targets in the cytosol resulting in activation of the Src/Ras/Erk pathway or modulation of nitric oxide. In addition, changes in metabolism of endogenous hormones, cross-talk between genomic and nongenomic pathways, cross talk with estrogen receptors after binding on other receptors, interference with feedback regulation and neuroendocrine cells, changes in DNA methylation or histone modifications, and genomic instability by interference with the spindle figure can play a role. Also it was found that effects of receptor activation can differ in function of the ligand.

## 1. Introduction

The objectives of the present paper include to give an overview of the wide spectrum of substances having endocrine-disrupting properties; to list a series of observations suggesting that endocrine disruptors contribute to human health problems, however, without attempting to bring an all-inclusive review for each individual substance accompanied by a final conclusion; to give an overview of the many mechanisms on which endocrine disruption rests.

## 2. Incidence and Prevalence of Health Problems associated with Endocrine Disruption Have Increased

Epidemiological data show increases in incidence and prevalence of diseases associated with endocrine-disrupting chemicals, such as breast, prostate, and testis cancer, diabetes, obesity, and decreased fertility over the last 50 years. A short overview of supporting data is presented below. These increases might partly reflect an increase in the likelihood of diagnosis and certainly do not constitute proof of the impact of endocrine-disrupting chemicals. Time trends and ecological studies are not well suited to study a possible association between exposure to endocrine disrupting chemicals and risk of disease, as assessment of exposure is extremely difficult. The data on time trends are, however, consistent with such an impact, as are data on the incidence or prevalence of some diseases in migrants and on differences in function of geographical area. Huge geographical differences in cancer incidence are well documented in the report “*Cancer incidence in five continents, volume IX*” of the International Agency for Research on Cancer [1], often showing higher risks of hormone-related cancers in more industrialized countries. In France, cancer mortality among migrants is positively associated with the Human Development Index of the country-of-birth [2]. In migrants to California, time since immigration is associated with convergence of the odds for mortality from cancer and from some other diseases to that of the native population [3]. In the Netherlands (for cancer mortality) [4], in British Columbia (for cancer incidence) [5], in the San Francisco Bay area (for risk of breast cancer) [6], and in Hawai (for cancer incidence among Japanese) [7], convergence towards the rates of the native population was reported. A systematic review and meta-analysis revealed that prevalence of obesity is higher among migrant Asian Indians than among Indians living in India [8]. In India, migration from rural to urban area's is associated with an increase in the prevalence of diabetes and obesity [9].

### 2.1. Cancer

**Figure 1:**
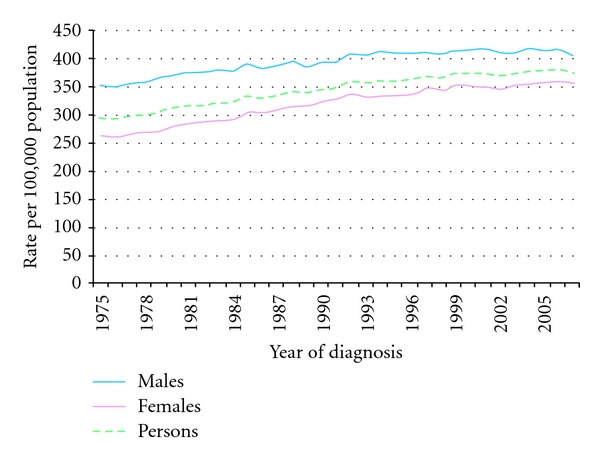
Age standardized (European) incidence rates for all cancers excluding nonmelanoma skin cancer, Great Britain 1975–2008. Figure taken from the web-site of the UK Cancer in Research organisation, accessed on 18/2/2011 [10].

In Great Britain, from 1978 to 2007, the overall age-standardized incidence rate of cancer has increased by 25%, an increase of 14% in men and a 32% increase in women [10] (see Figure 1). 

**Figure 2:**
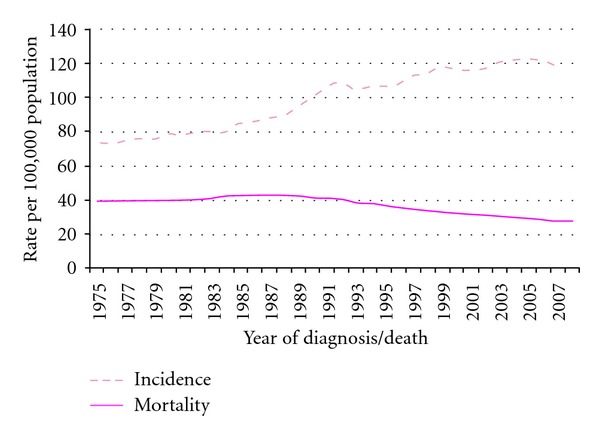
Age standardized (European) incidence and mortality rates for breast cancer in females in Great Britain 1975–2008. Figure taken from the web-site of the UK Cancer Research organisation, accessed on 18/2/2011 [10].

**Figure 3:**
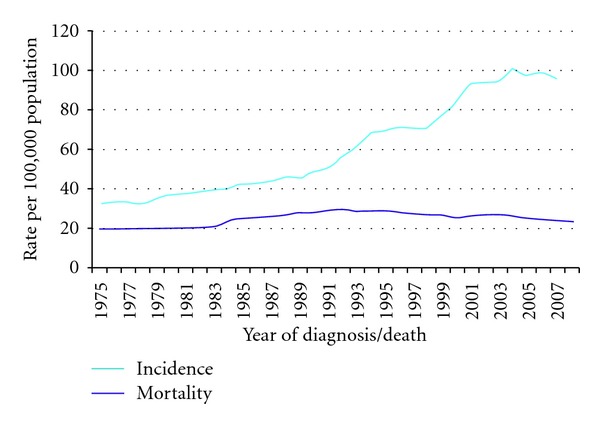
Age standardized (European) incidence and mortality rates for prostate cancer in males in Great Britain 1975–2008. Figure taken from the web-site of the UK Cancer Research organisation, accessed on 18/2/2011 [10].

According to Great Britain statistics, breast and prostate cancer incidence have a higher relative increase in incidence. Prostate cancer incidence has almost tripled from 33 per 100,000 in 1975 to 97 per 100,000 in 2007, while the incidence of breast cancer for women increased by 57%, from 77 per 100,000 in 1978 to 120 per 100,000 in 2007 (see Figures 2 and 3) [10]. These trends are being observed worldwide [11, 12]. Also the incidence of testis cancer has increased worldwide [13]. The increase in cancer incidence can partly be explained by the introduction of screening, which will result in a transient increase in incidence and a stage shift to earlier stages, microinvasive, and *in situ* cancers in the steady state situation [14]. It is, however very unlikely that early screening explains the entire rise in cancer incidence, because of the progressive nature of the cancer process, which leads almost always to clinically evident disease. Also, in the United States Incidence rates for all childhood cancers combined increased 0.6% per year from 1975 to 2002 [15], while cancer is a disease which, since many decades, is diagnosed more easily in children compared to elderly because of the particular impact of the disease on children. 

### 2.2. Diabetes

**Figure 4:**
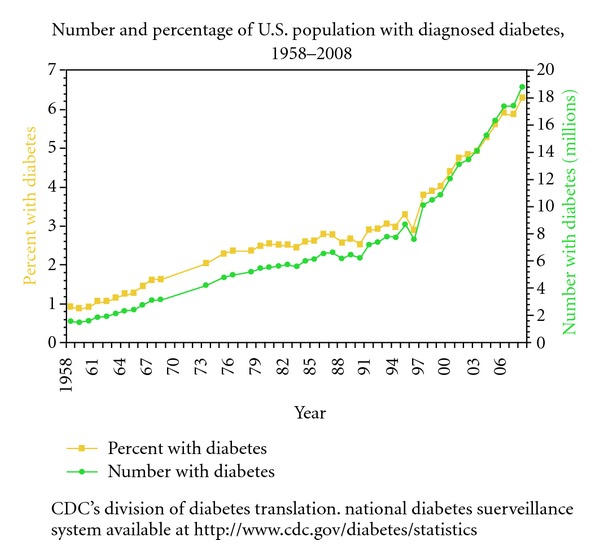
Number and percentage of US population with diagnosed diabetes 1958–2008, according to the CDC. Figure taken from the web-site of the Centers for Disease Control and Prevention, accessed on 18/2/2011 [16].

The United States Centre for Disease Control and Prevention (CDC) reports percentages of diabetes incidence of 0.93% in 1958 against 6.29% in 2008, which is a more than 6-fold increase, as is shown in Figure 4 [16].

### 2.3. Obesity

**Figure 5:**
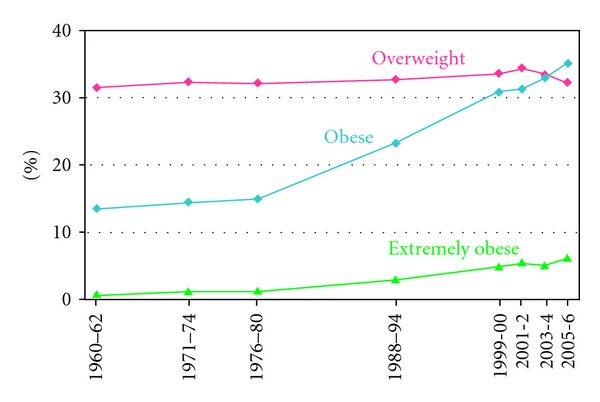
rends in overweight, obesity and extreme obesity over the last 50 years in the U.S, according to the CDC. Figure taken from the web-site of the Centers for Disease Control and Prevention, accessed on 18/2/2011 [17].

The CDC reports an increase of obesity prevalence (BMI ≥ 30) in US adults (20–74 years) from 13.4% in 1960–1962 to 35.1% in 2005-2006. Extreme obesity (BMI ≥ 40) has increased from 0.9% to 6.2% in the same period.  Figure 5 shows obesity trends among US residents over the last 50 years [17].

### 2.4. Metabolic Syndrome

In the United States, an increase in the prevalence of metabolic syndrome has been reported from 4.2% to 6.4% in adolescents, and from 23.1% to 26.7% in adults [18, 19].

### 2.5. Decreased Fertility

**Figure 6:**
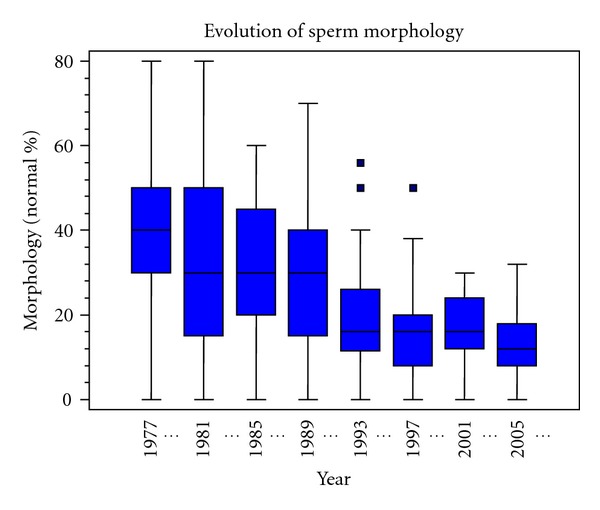
Sperm morphology for candidate sperm donors presenting themselves to the department of andrology of Ghent University, based on the data of Comhaire et al. [26].

In men fertility has been decreasing in the last decades, at least in some countries. Observations by Comhaire and colleagues [75] on candidate sperm donors show that this has been the case in Flanders (see Figure 6). Similar observations were made in Denmark [76], in France [77], and in the United Kingdom [78]. From a thorough analysis of 101 studies published 1934–1996 Swan et al. [79] concluded that sperm quality declined in the United States, Australia, and several countries in Europe. It seems likely that a decrease in semen quality plays a role in the recent decline in fertility rates [80]. Travison et al. [81] have recently reported declining levels of testosterone in US men of 1% per year, the same rate of decline seen for sperm concentrations. 

However, sperm quality is only one of several parameters determining human fertility. In terms of fertility of couples, in Sweden a transient increase in subfertility was seen in the early 1990s, but generally fecundability increased in Sweden between 1983 and 2002 [82]. As to ectopic pregnancies, in a Norwegian county population-based study age-adjusted ectopic pregnancy incidence rates increased from 4.3 to 16.0 per 10,000 women-years over the period 1970–1974 to 1990–1994 and declined to 8.4 per 10,000 women-years in 2000–2004 [83]. Joffe [84] found that couple fertility had increased in Britain, except for slight dips during 1976–80 and 1986–90.

Sallmén et al. [85] consider that, with the exception of rare settings in which the factors affecting reproductive choices have not changed, it is probably impossible to identify biologic changes in fertility of humans over recent decades.

The sex ratio (the ratio of the number of males born and the number of females) has decreased in many countries and most recently in the United States and Japanese populations [86]. 

## 3. Many Exposures Are associated with Endocrine Disruption

An endocrine-disrupting substance is a compound that alters the hormonal and homeostatic systems. Endocrine disruptors act via nuclear receptors, nonnuclear steroid hormone receptors (e.g., membrane ERs), nonsteroid receptors (e.g., neurotransmitter receptors such as the serotonin receptor, dopamine receptor, norepinephrine receptor), orphan receptors [e.g., aryl hydrocarbon receptor (AhR), an orphan receptor], enzymatic pathways involved in steroid biosynthesis and/or metabolism, and numerous other mechanisms that converge upon endocrine and reproductive systems. The most important aspects of endocrine disruption are related to xenoestrogens, antiestrogens, antiandrogens, disruption of thyroid function, and disruption of corticoid function, and other metabolic effects. 

The group of molecules identified as endocrine disruptors is highly heterogeneous and includes synthetic chemicals used as industrial solvents/lubricants and their by-products, plastic compounds, plasticizers, pesticides, pharmaceutical agents, and heavy metals such as cadmium and lead.

### 3.1. Xenoestrogens-, Xenoandrogens, Antiestrogens, and Antiandrogens

Many substances have estrogenic, androgenic, antiestrogenic, or antiandrogenic properties.  Table 1 shows a (certainly incomplete) list of such substances. Quite often the same substance has more than one of these properties. 

#### 3.1.1. Xenoestrogens

Many substances display estrogenic properties. These xenoestrogens (see Table 1) include phytoestrogen isoflavonoids, flavonoids and terpenoids and the mycotoxin zearalenone and its metabolites, and also many industrial chemicals [99]. Such man-made xeno-estrogens include polychlorinated biphenyls (PCBs); polybrominated biphenyl ethers (PBDEs), some endocrine disrupting derivatives of which occur also naturally [26]; phthalates; alkylphenols (degradation products of alkylphenolpolyethoxylates, surfactants used in cleaning detergents); bisphenol A, used in the production of polycarbonate plastic and epoxy resins; UV filters; the fragrance galaxolide; preservatives and pesticides. Of 200 pesticides tested for agonism to two human estrogen receptor (hER) subtypes, hERalfa and hERbeta by highly sensitive transactivation assays using Chinese hamster ovary cells, 47 and 33 showed hERalfa- and hERbeta-mediated estrogenic activities, respectively [63]. Several polycyclic aromatic hydrocarbons, mainly pollutants produced by incomplete combustion, were also shown to have estrogenic activity [100], for example, 3-Methylcholanthrene was shown to be estrogenic in MCF-7 human breast cancer cells [28]. Also, several metals were observed to have oestrogenic effects [101, 102], and certain cadmium-containing nanocrystals might even be potent estrogens [103]. 

Also complex mixtures of pollutants occurring in the environment were shown to have estrogenic activity. This was for instance shown for diesel exhaust particles [104].

Food constitutes the main exposure route for humans [99]. 

Xeno-oestrogens differ strongly in their oestrogenic potency, and assessing the health risks associated with exposure to xeno-estrogens is very complex [99]. 

#### 3.1.2. Xenoandrogens

Xenoandrogenic activity is less frequently reported than xenoestrogenic activity. There are, however, several authors describing xenoandrogenic activity (Table 1). For instance, Delor 103, a commercial mixture of PCB congeners, was reported to have androgenic activity in a bioluminescent yeast strain [51]. Using a Yeast androgen assay expressing the human androgen receptor, Kunz and Fent [55] tested 18 UV filters for androgenic activity and found that benzophenone-2 and homosalate produced full dose-response curves, while octyl-methoxycinnamate, octyl salicylate, octocrylene, and isopentyl-4-methoxycinnamate displayed a partly agonistic behaviour indicated by submaximal dose-response curves. 

#### 3.1.3. Antiestrogens

Many substances binding on estrogen receptors have antiestrogenic effects rather than estrogenic effects. In recent years, many substances (Table 1) and more complex exposures were found to have antiestrogenic activity on animals or in other experimental studies. Antiestrogenic effects were observed for methoxylated brominated diphenyl ethers (primarily of natural origin in the marine environment) (in reporter gene assays) [26]; 20S-protopanaxadiol, a major gastrointestinal metabolic product of ginsenosides (on MCF7 human breast cancer cells) [20]; genistein, a phytoestrogen, particularly abundant in soybeans that can bind estrogen receptors and sex hormone binding proteins, exerting (in vivo in animals) both estrogenic and antiestrogenic activity [25]; the polybrominated diphenyl ethers hepta-BDE and 6-OH-BDE-47 (in vitro) [43, 44]; the UV-absorber benzophenone-4 (in the liver of zebra fish) [58]; of 18 UV filters, 13 (4-Methylbenzylidene camphor, 3-Benzylidene camphor, Benzophenone-3, Benzophenone-4, Isopentyl-4-methoxycinnamate, Octyl-methoxycinnamate, Homosalate, Octocrylene, Benzyl salicylate, Phenyl salicylate, Octyl salicylate, Para amino-benzoic acid and Octyl dimethyl para amino benzoate) completely inhibited the activity of estradiol at the highest concentrations tested and produced full dose-response curves (in yeast carrying a human estrogen receptor), whereas one (Ethoxylated ethyl 4-amino benzoate) had a less pronounced activity [55]; polycyclic musks (on human U2OS cells with an estrogen receptor linked reporter gene) [60]; the di-ortho PCB congeners 38, 153, and 180 and the mono-ortho PCB 118 (on MCF-7-BUS cells in vitro) [52]; polychlorinated biphenyl 126 and phenanthrene (in the liver of fish) [53]; the pyrethroid insecticide metabolite 3-(2,2-dichlorovinyl)-2,2-dimethylcyclopropne carboxylic acid (DCCA), the pyrethroid insecticides cycloprothrin, etofenprox, the pyrethroid insecticide metabolite 3-phenoxybenzoic acid, the pyrethroid insecticides cyuflthrin and permethrin (in decreasing order, on reporter gene assays) [62]; the pyrethroid insecticide tetramethrin (on female rats in vivo) [74]; some nonylphenol isomers (on MVLN cells, MCF -7 human breast carcinoma cells with an estrogen receptor controlled luciferase reporter gene) [40]; dichlorostyrene (in the E-screen assay on MCF-7 cells) [38]; benzotriazole, an anticorrosive agent well known for its use in aircraft de-icing and antifreeze fluids but also used in dishwasher detergents (in a recombinant yeast estrogen assay, but not in vivo in adult fathead minnows) [34]. 

Also complex mixtures of pollutants occurring in the environment were shown to have antiestrogenic activity. This was observed (on yeast) for disinfection by-products formed during chlorination of waste water, especially 2,4-diphenylcrotonaldehyde, a relatively potent antiestrogenic chemical [113]; extracts from soils collected near highways (on MVLN cells) [114]; gaseous and particulate fractions of ambient air (on MVLN cells) [115]; extracts from sedimentation dust from subway stations (in yeast) [116]; extracts of motorcycle exhaust particulate (in MCF-7 human breast cancer cells and immature female rats) [117]; PAH mixtures in by-products of manufactured gas plant (MGP) residues [118]. Interestingly, some mixtures of polychlorinated biphenyls had antiestrogenic activity in MCF-7-BUS cells, whereas other mixtures, containing some of the same PCB congeners, had not [52]. 

The structural basis for estrogenic or rather antiestrogenic activities of polybrominated diphenyl ethers was studied by Yang et al. [119]. They showed, with docking studies on the human estrogen receptor alpha, that some of the PBDE compounds with antiestrogenic activity extended into the channel of the estrogen receptor (ER), which is usually occupied by the alkylamine side chain of the ER antagonists raloxifene and 4-hydroxytamoxifen, while most PBDE compounds without antiestrogenic activity adopted binding modes similar to that of ER agonist 17beta-estradiol (E2), located in the binding cavity and which did not protrude into the channel.

The omnipresence of antiestrogenic pollutants was demonstrated by Sanfilippo et al. [120] who, based on the yeast estrogen screen (YES) and chemical analysis (GC/MS), found antiestrogenic activity due to the presence of bis(2-ethylhexyl) phthalate (DEHP) in ultrapure water for laboratory use.

According to Bonefeld-Jorgensen [121], dioxins exert an antiestrogenic effect on Greenlandic Inuit. 

Some phytoestrogen, such as flavones and isoflavones, have antiestrogenic effects through inhibition of the aromatase enzyme converting testosterone to estradiol [122]. Conceivably, some man-made substances or pollutants might also have antiestrogenic effects through inhibition of the aromatase [123]. 

Estradiol inhibits gonadotropin release in men by an action at the hypothalamus and pituitary [124]. Higher internal exposure to antiestrogens might contribute, through inhibition of hypothalamic-pituitary feed-back mechanisms, to higher sex hormone concentrations. Recently, we found in some industrial areas in Flanders higher sex hormone concentrations in male adolescents (see reports on Flemish biomonitoring on http://www.milieu-en-gezondheid.be/onderzoek/luik%2021/hotspots/genkzuid/resultaten/STP%20MG%20Resultatenrapport%20Genk-Zuid%20-%20definitief.pdf, http://www.milieu-en-gezondheid.be/onderzoek/luik%2021/hotspots/menen/resultaten/STP%20MG%20eindrapport%20Menen%20DEF.pdf). 

#### 3.1.4. Antiandrogens

Many substances binding on androgen receptors have antiandrogenic effects rather than androgenic effects.  Table 1 shows some data on substances for which antiandrogenic effects were observed. Other data are mentioned below.

The polychlorinated biphenyl PCB#138 showed an antiandrogenic effect on androgen receptor activity in transiently cotransfected Chinese Hamster Ovary cells [54]. Using the stable prostatic cell line PALM, which contains a human androgen receptor (hAR) expression vector and the reporter MMTV-luciferase, Lemaire et al. [64] found several organochlorine pesticides to have antiandrogenic activity (Table 1). Of 18 UV filters tested, 16 substances (see Table 1) displayed antiandrogenic activity and showed full dose-response curves with complete inhibition of dihydrotestosterone activity in a Yeast androgen assay expressing the human androgen receptor [55]. The antiandrogenic activities of phenyl- and benzyl salicylate, benzophenone-1 and -2, and of 4-hydroxybenzophenone were higher than that of flutamide, a known hAR antagonist. For the DDT metabolite *Dichlorodiphenyldichloroethylene* (p,p′-DDE) and for 23 pesticides intensively used in Europe in recent years, antiandrogenic activity (Table 1) was reported [66]. The antiandrogenic activity of these pesticides was observed in vitro on MDA-kb2 cells, human breast cancer cells stably transfected with a firefly luciferase reporter gene that is driven by an androgen-response element-containing * *promoter [66]. Of 200 pesticides tested for antagonism to a human androgen receptor (hAR) by highly sensitive transactivation assays using Chinese hamster ovary cells, 66 of 200 pesticides exhibited antiandrogenic activity [63]. In particular, the antiandrogenic activities of two diphenyl ether herbicides, chlornitrofen and chlomethoxyfen, were higher than those of vinclozolin and p,p′-dichlorodiphenyl dichloroethylene, known AR antagonists [63]. The antiandrogenic activity of the organophosphorus pesticide fenthion was observed to be similar in magnitude to that of the antiandrogenic drug flutamide in an androgen-responsive element luciferase-reporter-responsive assay using NIH3T3 cells [71]. Whereas the insecticide permethrin might have estrogen-like effects on female rats, it showed antiandrogen-like effects on males [69]. Polycyclic musks were found to have antiandrogenic effects (on human U2OS cells with an androgen receptor linked reporter gene) [60]. Some methoxylated brominated diphenyl ethers (primarily of natural origin in the marine environment) were shown to be antiandrogenic in reporter gene assays [26].

Also complex mixtures of pollutants occurring in the environment were shown to have antiandrogenic activity. This was observed for some soils collected near highways (on MVLN cells) [114]; gaseous and particulate fractions of ambient air (on MVLN cells) [115]; diesel exhaust particles (in vivo in animals) [104].

### 3.2. Disruption of Thyroid Function

The thyroid hormone (TH) system with its main hormones tetraiodo-L-thyronine (T4) and the physiologically active 3,3,5-triiodo-L-thyronine (T3) are crucial regulators of many developmental processes (e.g., brain, inner ear, and bone development as well as bone remodelling) and physiological functions such as carbohydrate-, lipid-, and protein metabolism and homeostasis of the metabolic rate. Deficiencies can lead to important pathologies [125, 126]. Many chemical substances can contribute to disruption of the thyroid gland function, including PCBs [127, 128], the detergent derivative nonylphenol [126], the plasticiser dibutylphthalate [126], the plastic component bisphenol A [126, 129], some pesticides [126], the antibacterial agent triclosan [130], polybrominated biphenyls [131–133], perfluorinated compounds [134, 135], and some UV-filters [126]. Disruption of thyroid function happens to a large extent at the level of prereceptor regulation of ligand availability [125, 136, 137].

### 3.3. Disruption of Corticoid Function and Other Metabolic Effects

Endocrine disruption can also affect corticoid hormonal function, as observed for hexachlorobenzene in Wistar rats [138]. Hexachlorobenzene was also observed to induce oxidative stress, disruption of arachidonic acid metabolism, and porphyria [139, 140].

### 3.4. Activation of the Aryl Hydrocarbon Receptor

The aryl hydrocarbon receptor (AhR) regulates enzymes important to the metabolism of both endogenous substances (e.g., hormones) and exogenous substances, involved in both the detoxification and bioactivation of xenobiotics [143]. However, the AhR has been shown to regulate a much wider spectrum of cellular processes including cell proliferation, differentiation, apoptosis, and intercellular communication [144, 145]. TCDD, a prototype ligand to the AhR, is one of the most potent carcinogens [146] and designated by IARC as a human carcinogen. Activation of the AhR receptor probably plays an important role in tumorpromotion [144, 147]. Dioxins and other lipophylic dioxin-like substances constitute an important risk to human health through their presence in food items [148]. Also, activation of the AhR by polycyclic aromatic hydrocarbons might be a major toxic mode of action of air pollution particulate matter. Andrysik et al. [149] found that polycyclic aromatic hydrocarbons and their polar derivatives present in the organic fraction of air pollution were potent inducers of a range of AhR-mediated responses, including induction of the AhR-mediated transcription, such as cytochrome P450 1A1/1B1 expression, and the AhR-dependent cell proliferation. Importantly, these toxic events were observed at doses one order of magnitude lower than DNA damage. The AhR-mediated activity of the neutral fraction was linked to PAHs and their derivatives, as polychlorinated dibenzo-p-dioxins, dibenzofurans, and biphenyls, were only minor contributors to the overall AhR-mediated activity.

### 3.5. Internal Exposure to Endocrine Disrupting Chemicals Is Not Negligible

Andersen et al. [150] observed that occupational exposure to estrogen-like pesticides can result in detectable impacts on hormonal activity in the blood. Also, it has been shown that, in human placentas, xenoestrogenic activity amounted to 9.27% of the endogenous estrogenic activity in terms of the geometric mean, and to 9.84% in terms of the median value according to the MCF-7 breast cancer cell-based E-Screen [151]. Furthermore, Bonefeld-Jorgensen et al. [152] found that serum levels of the polychlorobiphenyl CB-153 and p,p′-DDE alone were not sufficient to predict xenoestrogenic serum activity, and that other xenoestrogens must have been present. In 2006, a bisphenol A (BPA) Expert Panel Consensus Statement was released by NIEHS/EPA, stating that most humans are exposed to BPA (95% of human urines are positive in the assay of the USA Center for Disease Control (CDC)). Unconjugated BPA in human serum is in the 0.3 to 0.5 ng/mL range, and the concentration of BPA in fetal serum, umbilical cord blood, amniotic fluid, and placenta indicate that the developing fetus is chronically exposed to BPA in the 0.7–9.2 ng/mL range (unconjugated BPA) [36].

## 4. Observations Indicating That Endocrine Disruptors Contribute to Health Deficits

### 4.1. Cancer

#### 4.1.1. Breast Cancer

Many observations point to the contribution of endocrine disruptors in the development of breast cancer. The 2009 Endocrine Society Scientific statement entails considerable evidence indicating that endocrine disruptors contribute to the risk of breast cancer [153].

**4.1.1.1. Experimental Studies**

Biological effects favouring malignant transformation were observed on human breast cells for: the pesticide hexachlorobenzene [154]; the organophosphorus pesticides malathion and parathion [155]; PCBs [156, 157]; bisphenol A [158, 159]; cadmium [160–162]; butyl benzyl phthalate [163]; organochlorine pesticides (p,p′-DDD plus p,p′-DDE plus o,p′-DDE plus aldrin plus dieldrin) [164]; cosmetics benzyl salicylate, benzyl benzoate and butylphenylmethylpropional (Lilial) [165]; nitrite [166].

The antiandrogenic pesticide vinclozolin induced, through epigenetic changes, a number of disease states or tissue abnormalities, including cancer of the breast, in adult rats from the F1 generation and all subsequent generations examined (F1–F4) [167].

Animal experiments showed that in utero exposure to dioxins induced alterations in breast development and increased susceptibility for mammary cancer development [168]. Mice [169] and rats [170] exposed perinatally to BPA showed preneoplastic lesions (intraductal hyperplasias) in mammary tissue and such rats developed carcinoma in situ. Rats exposed perinatally [171] or through lactation [172] to BPA showed an increased susceptibility to neoplastic development. Perinatal exposure to environmentally relevant levels of BPA induced an increase in the number of terminal end buds in the Mouse mammary gland and by 6 months of age, the mammary glands showed a dramatic expansion of the ductal network with a significant increase in terminal ducts and alveolar structures relative to the control [173]. Terminal end buds are the structures in which mammary cancer originates in both rodents and humans [174].

**4.1.1.2. Epidemiological Studies**

Table 2 resumes the main data from a number of epidemiological studies. Below some other data and additional details of some of these studies are mentioned.

López-Carrillo et al. [89] reported that exposure to diethyl phthalate may be associated with increased risk of breast cancer, whereas exposure to the parent phthalates of monobenzyl phthalate and mono (3-carboxypropyl) phthalate might be negatively associated.

In a prospective study on young women, from Oakland, California, who provided blood samples in the period 1959–1967, high levels of serum p,p′-DDT predicted a statistically significant 5-fold increased risk of breast cancer among women who were born after 1931 [98]. These women were under 14 years of age in 1945, when DDT came into widespread use, and mostly less than 20 years.

DDT use peaked. These findings suggest that exposure to p,p′-DDT early in life may increase breast cancer risk [98]. In a systematic review and meta-analysis of studies concerning cyclodiene insecticides and breast cancer, Khanjani et al. [175] found a significant association between heptachlor and breast cancer, but no significant association with other cyclodiene insecticides. Mills and Yang [176] performed a registry-based case-control study of breast cancer in farm labor union members in California. Controlling for covariates, adjusted ORs (and 95% CIs) for breast cancer in quartiles of pesticide use were 1.00, 1.30 (0.73–2.30), 1.23 (0.67–2.27), and 1.41 (0.66–3.02). Chlordane, malathion, and 2,4-D were associated with increased risk. Risk associated with chemical use was stronger in younger women, those with early-onset breast cancer, and those diagnosed earlier [176].

#### 4.1.2. Prostate Cancer

**4.1.2.1. Experimental Studies**

As noted by Gail Prins [177], who contributed importantly to the study of prostate carcinogenesis, there is substantial evidence from animal models that specific endocrine-disrupting compounds may influence the development or progression of prostate cancer. In large part, these effects appear to be linked to interference with estrogen signaling, either through interacting with ERs or by influencing steroid metabolism and altering estrogen levels within the body. Studies in animal models show augmentation of prostate carcinogenesis with several environmental endocrine disruptors including diethylstilboestrol, PCBs, cadmium, ultra violet filters, Bisphenol A, and arsenic. Importantly, there appears to be heightened sensitivity of the prostate to these endocrine disruptors during the critical developmental windows including in utero and neonatal time points as well as during puberty. Thus infants and children may be considered a highly susceptible population for endocrine-disrupting exposures and increased risk of prostate cancers with aging [177].

Bisphenol A, a plastic component that can be considered a model agent for endocrine disruption, was shown to induce changes in differentiation patterns, cell proliferation, and size in the prostate, changes that are probably associated with an increase in cancer risk [178].

Exposure to diethylstilbestrol (DES), especially prenatal and early in life, has been associated with prostate abnormalities, including prostatic squamous neoplasia [177].

UV-filters, compounds of sunscreens, have been reported to alter prostate gland development and estrogen target gene expression in rats. Especially 4-methylbenzylidene and 3-benzylidene-camphor are ERbeta ligands [179–181].

Cadmium is a known ER ligand. In vitro work has shown proliferative action of cadmium in human prostate cells, and in rats prostatic tumors have been induced by oral cadmium exposure or by injection [177, 182, 183].

According to a review by Benbrahim and Waalkes, arsenic can induce malignant transformation of human prostate epithelial cells and also appears to impact prostate cancer cell progression by precipitating events leading to androgen independence in vitro [184].

Vinclozolin, a fungicide, has known antiandrogenic properties. On the one hand prostate gland growth in rats was reduced by vinclozolin, but on the other hand, prenatal exposure leads to aging-associated prostatitis in the next four generations of offspring. This suggests a role in prostate cancer induction [177].

**4.1.2.2. Epidemiological Studies**

Table 3 resumes the main data from a number of epidemiological studies. Below some other data and additional details of some of these studies are mentioned.

The Agricultural Health study in Iowa and North Carolina showed that Farmers and commercial pesticide applicators have a significantly increased risk of prostate cancer (http://www.aghealth.org/).

Kumar et al. [185] studied 70 newly diagnosed prostate cancer patients and 61 age-matched healthy male controls. Significantly higher levels of betahexachlorohexane, gamma-hexachlorohexane, and p,p-DDE were found in cases as compared to controls, with increases of 32%  (*P* = 0.04), 38%  (*P* = 0.008), and 38%  (*P* = 0.01), respectively.

Among the participants in the Agricultural Health Study in Iowa and North Carolina, different categories of use of the fumigant methyl bromide in terms of percentiles of use (<33.3, 33.4–66.7, 66.8–83.3, 83.4–91.6, >91.6), the odds ratio's (95% C.I.) amounted, compared to participants without use, to 1.01 (0.66–1.56), 0.76 (0.47–1.25), 0.70 (0.38–1.28), 2.73 (1.18–6.33), 3.47 (1.37–8.76) after correction for age and family history of prostate cancer [110]. In the same study, different categories of use of chlorinated pesticides, among applicators over 50 years of age, in terms of percentiles of use (<33.3, 33.4–66.7, 66.8–83.3, 83.4–91.6, >91.6), the odds ratio's (95% C.I.) amounted, compared to participants with a use below percentile 33.3, to 1.29 (1.02–1.63), 1.51 (1.15–2.00), 1.37 (0.96–1.97), 1.39 (0.99–1.97), after correction for age and family history of prostate cancer [110]. Still in the same study, the thiocarbamate herbicide butylate, the organophosphorothioate insecticides chlorpyrifos and coumaphos, the organophosphorodithioate pesticide fonofos, the pyrethroid insecticide permethrin, and the organophosphorodithioate insecticide phorate showed a significantly increased risk of prostate cancer among study subjects with a family history of prostate cancer but not among those with no family history [110, 186].

#### 4.1.3. Testis Cancer

In the recent past, there have been several new epidemiological papers (reviewed by Aitken et al. [187] that point towards widespread declines in sperm quality and increases in testis cancer incidence and suggesting that these likely have an endocrine etiology. Skakkebæk et al. [188] hypothesized that the testicular dysgenesis syndrome originates from conception and can result in a cascade of defects in Sertoli and Leydig cells that ultimately affect maldescent of the testes, cryptorchidism, fertility, and the probability of testicular cancer. Jorgensen et al. [189] observed that the increasing incidence of testis cancer in Finnish birth cohorts was associated with a substantial decrease in semen quality and concluded that these simultaneous and rapidly occurring adverse trends suggested that the underlying causes are environmental and, as such, preventable.

#### 4.1.4. Non-Hodgkin Lymphoma and Other Hematopoietic Cancers

Table 4 resumes the main data from epidemiological studies. Below some other data and additional details of some of these studies are mentioned.

Merhi et al. [190] performed a meta-analysis of 13 case control studies that examined the occurrence of hematopoietic cancers in pesticide-related occupations. The overall meta-odds ratio obtained after pooling 44 ORs from 13 studies was 1.3 (95% CI: 1.3–1.5). In particular, a significant increased risk of NHL was found (OR = 1.35; 95% CI = 1.2–1.5). 

### 4.2. Diabetes

According to Harrison's Principles of Internal Medicine (16th edition), diabetes is a metabolic disease caused by an attenuated production of insulin by pancreas B-cells (type I diabetes) or by development of resistance against insulin action resulting in a relative insulin-shortage (type II diabetes).

#### 4.2.1. Experimental Data

According to a review by Alonso-Magdalena et al. [203], widespread EDCs, such as dioxins, pesticides and bisphenol A, cause insulin resistance and alter *β*-cell function in animal models. Many of them act as estrogens in insulin-sensitive tissues and in *β* cells, generating a pregnancy-like metabolic state characterized by insulin resistance and hyperinsulinemia.

Adult male Sprague-Dawley rats exposed to crude salmon oil, containing persistent organic pollutants (POPs), developed insulin resistance, abdominal obesity, and hepatosteatosis. The contribution of POPs to insulin resistance was confirmed in cultured adipocytes where POPs, especially organochlorine pesticides, led to robust inhibition of insulin action [204].

Parathion and other organophosphate pesticides induce a prediabetic state in Sprague-Dawley rats in a sex-selective manner [205].

Long-term exposure to the endocrine disrupting herbicide atrazine induces morphological and functional changes in mitochondria, a decrease in metabolic rate, insulin resistance and obesity in Sprague-Dawley rats [206].

The xenoestrogen Bisphenol A disrupted the action of the endocrine pancreas and blood sugar homeostasis in mice: a postprandial hyperinsulinemia and insulin resistance were induced after an injection of 100 *μ*g/kg per day during 4 days in mice [207].

In Wistar rats, prenatal exposure to diisobutyl phthalate reduced plasma leptin and insulin in male and female fetuses [208].

Arsenic inhibits, in 3T3-L1 adipocytes in vitro, insulin signaling by inhibiting the PDK-1/PKB/Akt signal transduction pathway, which might explain its diabetogenic effects [209].

#### 4.2.2. Epidemiological Studies

Table 5 resumes the main data from a number of epidemiological studies. Below some other data and additional details of some of these studies are mentioned.

**4.2.2.1. Dioxins**

As mentioned by Lee et al. [196], the U.S. Department of Veterans Affairs added type 2 diabetes to the list of presumptive diseases associated with the exposure to dioxin-containing Agent Orange in Vietnam.

Inhabitants of Seveso who were exposed to very high concentrations of dioxin during the disaster in 1976 showed significantly more diabetes than nonexposed inhabitants [191].

Longnecker and Daniels [210] identified 11 reports on the relation of TCDD with type 2 diabetes, 5 of which showed an unequivocally positive association with 3 others showing an equivocally positive association, but all these studies showed notable weaknesses.

Among 69 subjects in good health residing within 25 miles of the Vertac/Hercules Superfund site in Jacksonville, Arkansas, serum lipid concentration of TCDD ranged between 2 and 94 ppt [194]. Plasma insulin concentrations (*μ*IU/mL) at fasting and 30, 60, and 120 minutes after a 75 g glucose load were significantly (*P* < 0.05) higher in persons with top decile TCDD levels (>15 ppt), respectively 7.0 ± 8.4 versus 2.0 ± 2.5, 412 ± 780 versus 79 ± 113, 325 ± 317 versus 100 ± 159, and 294 ± 431 versus 65 ± 166 [194].

After adjustment for age and other covariates, serum total toxic equivalent activity (sum of PCDD/Fs and coplanar PCBs) was 62% (*P* = 0.0005) higher in diabetic patients, than in controls [192].

**4.2.2.2. Other POPs (Mainly Organochlorine)**

After adjustment for age and other covariates, concentration of 12 marker PCBs was 39% (*P* = 0.0067) higher in diabetic patients than in controls [192].

In a cross-sectional study of 2,245 pregnant women, of whom 44 had diabetes (primarily type 1), the adjusted mean serum level of PCBs among the subjects with diabetes was 30% higher than in the control subjects (*P* = 0.0002), and the relationship of PCB level to adjusted odds of diabetes was linear [193].

According to the observations of Lee et al. [197], the dioxin-like PCBs and the organochlorine pesticides showed the strongest associations with diabetes.

In a nested case-control within the Coronary Artery Risk Development in Young Adults (CARDIA) cohort, Lee et al. [197] measured 8 organochlorine pesticides, 22 polychlorinated biphenyl congeners (PCBs), and 1 polybrominated biphenyl (PBB) in serum collected in 1987-1988. Participants in this nested case-control study were diabetes free in 1987-1988. By 2005-2006, the 90 controls remained free of diabetes, whereas the 90 cases developed diabetes. POPs showed nonlinear associations with diabetes risk. The highest risk was observed in the second quartiles of trans-nonachlor, oxychlordane, mirex, highly chlorinated PCBs, and PBB153, a finding that suggests low-dose effects. Sextiles of a summary measure for all 31 pops measured were associated with following odds ratios after adjustment for wet-weight model, adjusted for age, sex, race, and BMI: sextile1 reference; 2.9 (1.0–8.8); 4.8 (1.5–14.8); 1.5 (0.4–4.8); 1.9 (0.6–4.8); 2.7 (0.8–8.8).

Lee et al. [196] reported strong and highly significant associations, among participants in the NAHNES study, between serum concentrations of persistent organic pollutants and the prevalence of diabetes, with high Odds ratio's (Table 5) after correction for age, sex, race and ethnicity, poverty, income, BMI, and waist circumference. In nondiabetic adults participating in the same NAHNES study, successive quartiles of a parameter for sum of serum concentrations of organochlorine pesticides (Oxychlordane, Trans-nonachlor, p,p-dichlorodiphenyltrichloroethane and -Hexachlorocyclohexane) were associated with HOMA-IR insuline resistance values of 3.27 ± 0.39, 3.36 ± 0.33, 3.48 ± 0.35, and 3.85 ± 0.45 (*P*_trend_ < 0.01) [198], after adjustment for age, sex, race, poverty income ratio, BMI, waist circumference, cigarette smoking, serum cotinine concentration, alcohol consumption, and exercise. This association increased in power in function of increasing HOMA-IR values: correlated odds-ratio for the comparison of the highest and the lowest quartile of serum concentration of organochlorine pesticides were 1.8 for an insulin resistance value equal to or higher than the 50th percentile, 4.4 for the 75th percentile and 7.5 for the 90th percentile. This means that, when comparing high and low exposed individuals, the odds-ratio for a strong abnormal insulin resistance is clearly higher than for a weakly increased insulin resistance [198].

The associations found between some PCBs and organochlorine pesticides with insulin resistance among subjects without diabetes [198] are consistent with the notion that these substances contribute to the induction of diabetes. When all five subclasses of POPs were included in one model, only OC pesticides were significantly associated with insulin resistance [198].

The rather high correlation between serum levels of different organochlorine pollutants can lead to noncausal associations between some organochlorines and diabetes, simply because these organochlorines are themselves correlated with other organochlorines, which do have a causal association with diabetes.

Interestingly, in the National Health and Nutrition Examination Survey, obesity did not increase the prevalence of diabetes among subjects with nondetectable levels of POPs, even though there were sufficient numbers of study subjects at risk in each BMI category [196].

Everett et al. [211] argue that the probability of a causal association between PCBs and diabetes is weakened by the fact that many observations fail to show a linear dose-effect relationship. The existence of nonlinear and nonmonotonic effects is, however, to be expected for receptor-binding substances (see Section 5.2).

**4.2.2.3. Brominated Flame Retardants**

Lim et al. [199] studied levels of brominated flame retardants in relation to the prevalence of diabetes. Adjusted odds ratios across quartiles of serum concentrations for polybrominated biphenyl 153 (PBB-153) or polybrominated diphenyl ether 153 (PBDE-153) were 0.7 (0.3–1.6), 1.4 (0.7–3.0), 1.6 (0.8–3.5), and 1.9 (0.9–4.0) (*P*_trend_ < 0.01) and 1.6 (0.7–3.6), 2.6 (1.2–5.8), 2.7 (1.2–6.0), and 1.8 (0.8–4.0) (*P* for quadratic term <0.01), respectively. PBDE-153 thus showed an inverted U-shaped association with diabetes. Although both PBDE-99 and PBDE-100 tended to show inverted U-shaped associations similar with PBDE-153, they failed to reach statistical significance. Serum concentrations of PBB-153 were not associated with those of PBDEs, except for a weak correlation with PBDE-153. All five PBDEs were strongly and positively associated among each other [199].

**4.2.2.4. Phthalates**

Among 221 adult Mexican women, participants with diabetes (*n* = 39) had significantly higher concentrations (geometric means ± SD) of di(2-ethylhexyl) phthalate (DEHP) metabolites (213.4 ± 2.1 versus 161.6 ± 2.0) but lower levels of monobenzyl phthalate (MBzP) a metabolite of benzylbutyl phthalate (3.8 ± 3.9 versus 7.0 ± 2.9), compared to participants without diabetes [200]. Borderline significant increased risks for diabetes were observed in relation to DEHP metabolites except MEHP in contrast to the decreased risk that resulted with MBzP concentration [200].

Stahlhut et al. [212] studied 622 (only 327 for MEHHP and MEOHP) adult US male participants in the NHANES 1999–2002 to evaluate six phthalate metabolites with prevalent exposure and known or suspected antiandrogenic activity as predictors of insulin resistance measured through the log-transformed homeostatic model assessment (HOMA, a measure of insulin resistance). Using multiple linear regression, adjusted for age, race/ethnicity, fat and total caloric consumption, physical activity level, serum cotinine, and urine creatinine, urinary concentrations of three metabolites (log transformed to normalize the data) were associated (regression coefficient (SE), *P*), with increased insulin resistance: MBP (0.064 (0.024), *P* = 0.011), MBzP (0.079 (0.023), *P* = 0.002), and MEP (0.056 (0.020), *P* = 0.008). Converting regression coefficients to clinically interpretable measures, an increase in phthalate metabolites from the 10th to the 90th percentile corresponded to an increase in HOMA (at the 2.50 median) with 1.3-1.4 (52–57% of median) in association with three metabolites: MBP (1.3), MEP (1.3), and MBzP (1.4).

**4.2.2.5. Arsenic**

Epidemiologic evidence points to an increased chance for the development of type 2 diabetes in populations exposed to arsenic. These associations were, however, only found at relatively high, occupational exposures, or in areas with very high drinking water concentrations of arsenic, such as parts of Bangladesh and Taiwan [210]. According to Longnecker [213], more studies are needed on the association of diabetes with arsenic. Higher cadmium, arsenic, and lead levels have also been found in diabetic mothers compared to controls [214].

**4.2.2.6. Tentative Conclusion**

The associations between exposure to xenobiotics and diabetes in epidemiological studies are quite complex, and it is still difficult to establish a causal relation between a specific chemical and diabetes in humans, amongst others because clear dose-effect relationships are often lacking. However, taking into account the results of animal experiments, the consistency between epidemiological findings as to diabetes, insulin resistance, and the metabolic syndrome (see Section 4.4) and the mechanistic data suggesting that nonlinear and nonmonotonic exposure-effect relations are to be expected (see Section 5.2), it is likely that some organochlorines (especially some pesticides), some brominated chemicals, phthalates, and high levels of exposure to arsenic contribute to the risk of diabetes.

### 4.3. Obesity

Fat soluble xenobiotics can accumulate in adipose tissue, which then functions as a reservoir from which these substances can return to the blood, where they are detected in concentrations that show, for some xenobiotics-a positive correlation to the body mass index [215]. Irigaray et al. [216] observed that benzo(a)pyrene can induce obesity in mice by inhibition of beta-adrenergic stimulation of lipolysis in adipose tissue. However, negative correlations are often observed between body mass index and serum levels of organochlorine pollutants, probably due to a dilution effect. In Flemish adolescents, we found a significant negative association between internal organochlorine exposure (especially marker PCBs and hexachlorobenzene) and body mass index [217], negative association that might in part be explained by a dilution effect, but that might also, at least partly, result from an endocrine disruption effect suggesting that some organochlorines might, at least in adolescents, have a limiting effect on weight gain.

Several studies also suggest a role in obesity for the endocrine disrupting organotins tributyltin (TBT) and triphenyltin (TPT). TBT and TPT are potent agonists of the nuclear hormone receptors peroxisome proliferator-activated receptor gamma and retinoid X receptor, which serve as metabolic sensors for lipophilic hormones, dietary fatty acids and their metabolites, and consequently influence lipid biosynthesis and storage [218, 219].

Phthalates are known to act as PPAR activators, thyroid hormone axis antagonists or antiandrogens, and are suspected obesogens [219]. Several phthalate metabolites were positively and significantly correlated with abdominal obesity in a large US study [212, 220]. Hatch et al. [220] found positive associations in males (age 20–59) between urinary phthalate metabolite levels and BMI and waist circumference across quartiles of mono-benzyl (MBzP) phthalate (adjusted mean BMI = 26.7, 27.2, 28.4, 29.0, *P*_trend_ = 0.0002), and positive associations were also found for mono-2-ethyl-5-oxohexyl- (MEOHP), mono-2-ethyl-5-hydroxyhexyl- (MEHHP), mono-ethyl- (MEP), and mono-n-butyl- (MBP) phthalate. In females, BMI and WC increased with MEP quartile in adolescent girls (adjusted mean BMI = 22.9, 23.8, 24.1, 24.7, *P*_trend_ = 0.03), and a similar but weaker pattern was seen in 20–59 year olds. In contrast, MEHP was inversely related to BMI in adolescent girls (adjusted mean BMI = 25.4, 23.8, 23.4, 22.9, *P*_trend_ = 0.02) and females aged 20–59 (adjusted mean BMI = 29.9, 29.9, 27.9, 27.6, *P*_trend_ = 0.02). There were no important associations among children, but several inverse associations among 60–80 year olds. Effects were thus most pronounced among adults (20–59 years) and several phthalate metabolites were sex specifically associated [220]. Interestingly, we found, in the Flemish biomonitoring, sex-specific associations between urinary phthalate metabolite concentrations and sexual maturation (unpublished results). 

Polybrominated diphenyl esters (PBDE's) are potential obesogens as well. In a rat study PBDE's were found to lower thyroxin levels and affect lipolysis and insulin stimulated glucose oxidation in isolated adipocytes [221]. Low-dose exposure to PBDE's during gestation and lactation has also been observed to cause long-term changes in thyroid gland morphology [218].

Dithiocarbamates (present in cosmetics and pesticides) are suggested obesogens, through interference with glucocorticoid receptor signaling [219].

Perfluorooctanoic acid (PFOA) might also act as an obesogen under certain circumstances [222].

Heindel and vom Saal [223] have also reviewed effects of perinatal exposure to several endocrine disruptive chemicals on homeostatic control systems required to maintain normal body weight troughout life. These chemicals include cadmium, organotins, and bisphenol-A. 

### 4.4. Metabolic Syndrome

Table 6 resumes the main data from a number of epidemiological studies. Below some other data and additional details of some of these studies are mentioned.

The metabolic syndrome is characterized by a high blood pressure, hyperglycemia and hypertriglyceridemia, low HDL concentrations and too much fat in the waist area.

#### 4.4.1. Organochlorines

Lee et al. [224] found a correlation between serum concentrations of persistent organic pollutants and the prevalence of the metabolic syndrome in nondiabetic adults. Organochlorine pesticides correlated most strongly, with corrected odds ratios of 1.0, 1.5, 2.3, and 5.3 for the OC pesticide quartiles. Dioxin-like PCB's also showed a positive correlation with ORs 1.0, 1.1, 2.2, and 2.1. Non-dioxin-like PCBs showed an inverted-U association, with odds ratio's of 1.0, 1.3, 1.8, and 1.0 (*P* for quadratic term <0.01). OC pesticides showed ORs >2 for four of the five components of metabolic syndrome: waist circumference, elevated triacylglycerol, low HDL-cholesterol, and high fasting glucose. PCBs were linearly or quadratically associated with three of the five components: waist circumference, elevated triacylglycerol, and high fasting glucose. PCDDs and PCDFs were not associated with 4 of the 5 metabolic syndrome parameters but were positively and significantly associated with high blood pressure.

In a cross-sectional study on 1,374 subjects, not occupationally exposed to dioxins and related compounds, representative of the general population in Japan, the toxic equivalents (TEQs) of PCDDs, PCDFs, and DL-PCBs and total TEQs had significant adjusted associations with metabolic syndrome. The DL-PCB TEQs and total TEQs were associated with all components of the syndrome, and the odds ratios (ORs) in the highest quartile of DL-PCB TEQs in four of the five components were higher than those for PCDDs or PCDFs. Also congener-specific associations with metabolic syndrome were observed; in particular, the highest quartiles of PCB-126 and PCB-105 had adjusted ORs of 9.1 and 7.3, respectively [225].

#### 4.4.2. Brominated Flame Retardants

Brominated flame retardants have also been associated with the metabolic syndrome. Prevalence was positively correlated to PBB-153 in a study of Lim et al. [199], with PBB-153 quartile odds ratios 1.0, 1.5, 3.1, 3.1, and 3.1 (*P* for trend <0.01). PBDE-153 showed an inverted U-shaped association with metabolic syndrome with odds ratios of 2.1, 2.5, 2.4, and 1.6. Nonmonotonic dose-effect relations are to be expected for some receptor-binding substances (see Section 5.2).

#### 4.4.3. Phthalates

Stahlhut et al. [212] studied 1,292 (for MEHHP and MEOHP only 696) adult US male participants in the National Health and Nutrition Examination Survey (NHANES) 1999–2002 to evaluate six phthalate metabolites with prevalent exposure and known or suspected antiandrogenic activity as predictors of waist circumference. Using multiple linear regression, adjusted for age, race/ethnicity, fat and total caloric consumption, physical activity level, serum cotinine, and urine creatinine, urinary concentrations of four metabolites (log transformed to normalize the data) were associated (regression coefficient (SE), *P*), with increased waist circumference: MBzP (1.29 (0.34), *P* = 0.001), MEHHP (1.71 (0.56), *P* = 0.008), MEOHP (1.81 (0.60), *P* = 0.009), and MEP (0.77 (0.29), *P* = 0.013). Converting regression coefficients to clinically interpretable measures, an increase in phthalate metabolites from the 10th to the 90th percentile corresponded to an increase in waist circumference of 3.9 to 7.8 cm (4.0–8.0% of the 97.0-cm median) for four significant metabolites: MEP (3.9 cm), MBzP (5.8 cm), MEHHP (7.3 cm), and MEOHP (7.8 cm).

#### 4.4.4. Perfluorinated Compounds

Data from NHANES and from the C8 Health Project, mentioned in a recent review on perfluorooctanoic acid [226], suggest that perfluorooctanoic acid (PFOA) contributes to higher blood lipid concentrations. 

### 4.5. Infertility

#### 4.5.1. In Animals

Serious problems of reproductive health in wildlife caused by pesticides were reported in the important book “Silent Spring” [227]. Later work on the reproductive and developmental anomalies in Great Lakes gulls [228] and in alligators [229] and advances in understanding of the mechanism of action of diethylstilbestrol and other xenoestrogens [230] contributed to the formulation of the endocrine disruptor hypothesis [231]. Recent studies have documented disturbing reproductive effects of ambient levels of the pesticide atrazine on frogs, characterised by feminization of males that had been exposed to concentrations that can be encountered through permitted uses in the United States [232]. 

There are increasing data from wildlife studies and laboratory studies with rodents, ungulates, and nonhuman primates that support a role of EDCs in the pathogenesis of several female reproductive disorders, including polycystic ovarian syndrome, aneuploidy, premature ovarian failure (POF), reproductive tract anomalies, uterine fibroids, endometriosis, and ectopic gestation [153]. In sheep and rhesus monkeys, prenatal androgenic stimulation gives rise to the polycystic ovary syndrome. In rats, exposure to 2,3,7,8-tetrachlorodibenzo-p-dioxin (TCDD) in utero and through the end of reproductive life results in a dose-dependent onset of premature reproductive senescence, likely due to direct effects on ovarian function [233]. Perinatal exposure of rats to Bisphenol A affects the fertility of male offspring [234]. 

#### 4.5.2. In Humans

**4.5.2.1. Changes in Sex Ratio**

In a small First Nation Chippewa community surrounded by chemical manufacturing plants in Sarnia, Ontario, Canada, a marked decline in the sex ratio was observed, inferring that almost 40% of the boys had been lost [235]. In Seveso, Italy, a 2,4,5, trichlorophenol explosion in 1976 resulted in an immediate loss of males with subsequent recovery [236]. Van Larebeke et al. [237] proposed that sex ratio changes might constitute sentinel health events of endocrine disruption.

**4.5.2.2. Fertility of Women**

Daughters of women exposed to Diethylstilboestrol showed a reduced fertility [238].

According to the endocrine society scientific statement, bisphenol A and other endocrine disrupting chemicals might contribute to the risk of polycystic ovary syndrome (PCOS) in women [153]. PCOS is a debilitating disorder in women, occurring in 6.6% of the reproductive age population; it is a leading cause of subfertility and is associated with increased lifetime risks of cardiovascular disease and type II diabetes [153].

Adult exposure in women to cigarette smoke results in decreased fecundity, decreased success rates of in vitro fertilization (IVF), decreased ovarian reserve, earlier menopause by 1–4 years, and an increased miscarriage rate [239, 240]. Maternal smoking during pregnancy appears to be a threat to the future fecundity of the daughter [241].

Higher PCB serum levels were reported to be associated with increasing menstrual cycle length and a trend towards irregular cycles [242].

Among 50 Southeast Asian immigrant women, after correction for confounding, mean luteal phase length was shorter by approximately 1.5 days at the highest quartile of serum DDT (95% CI = −2.6 to −0.30) or DDE (−2.6 to −0.20). Progesterone metabolite levels during the luteal phase were consistently decreased with higher DDE concentration [243]. Cohn et al. [244] investigated time to pregnancy (fecundability), a sensitive indicator of environmental effects on reproduction, among 289 women exposed to p,p′-DDT and p,p′-DDE in utero. Maternal p,p′-DDE was associated with a raised probability of pregnancy in their daughters, and p,p′-DDT was associated with a reduced probability of pregnancy in daughters.

Among 1240 women from the Danish National Birth Cohort recruited from 1996 to 2002, longer “time to pregnancy” (TTP) was associated with higher maternal levels of PFOA and PFOS (*P* = 0.001) [245]. Compared with women in the lowest exposure quartile, the adjusted odds of infertility (defined as TTP > 12 months) increased by 70–134% and 60–154% among women in the higher three quartiles of PFOS and PFOA, respectively. Fecundity odds ratios (FOR) were virtually identical for women in the three highest exposure groups of PFOS (FOR (1/4) 0.70, 0.67, and 0.74, resp.) compared with the lowest quartile. A linear-like trend was observed for PFOA (FOR 0.72, 0.73, and 0.60 for three highest quartiles versus lowest quartile) [245].

Endometriosis, occurring in 6 to 10% of women, is associated with infertility. In utero exposure of humans to diethylstilboestrol results in an increase in relative risk of endometriosis (RR = 1.9, 95% confidence interval, 1.2–2.8) [246].

Also phthalates might contribute to the risk of endometriosis. Endometriotic women showed significantly higher plasma DEHP phthalate concentrations than controls (median 0.57 micro g/mL, interquartile range: 0.06–1.23; values range: 0–3.24 versus median 0.18 micro g/mL, interquartile range: 0–0.44; values range: 0–1.03; *P* = 0.0047) [247]. In a study in India, 49 infertile women with endometriosis showed significantly higher blood concentrations of di-n-butyl phthalate (DnBP), butyl benzyl phthalate (BBP), di-n-octyl phthalate (DnOP), and diethyl hexyl phthalate (DEHP) (mean 0.44 (SD 0.41); 0.66 (SD 0.61); 3.32 (SD 2.17); 2.44 (SD 2.17) micrograms/mL) compared with 21 age-matched women with proven fertility and no evidence of endometriosis (mean 0.15 (SD 0.21); 0.11 (SD 0.22); not detected; 0.45 (SD 0.68) micrograms/mL) [248].

Flemish mothers with higher concentrations of dioxin-like substances, PCBs, or hexachlorobenzene in cord blood at delivery were more likely to have undergone treatment for infertility with odds ratio's (95% C.I.) equal to, respectively, 1.40 (1.09–1.80,*P* = 0.008), 1.29 (1.04–1.59, *P* = 0,02), and 1.21 (1.01–1.45,*P* = 0.04) after correction for age and smoking (results from our Flemish biomonitoring program, see http://www.milieu-en-gezondheid.be/resultaten/2001-2006/pasgeborenen/UitgebreidResultatenrapport.pdf).

**4.5.2.3. Fertility of Men**

 

**(i) Maternal Smoking**

Maternal smoking during pregnancy appears to be a threat to the future fecundity of the son [249, 250].

**(ii) Dioxin Incidents**

Men (*n* = 40) exposed to PCBs and PCDFs during the “Yu-Cheng” incident were found to have higher abnormal morphology (27.5 (9.4)% versus 23.3 (5.3)%, *P* = 0,04) and oligospermia rate (22,5% versus 1%, *P* = 0,04) than controls [251]. The ability of sperm to penetrate hamster oocytes (% oocytes penetrated 16,2 (14,0) versus 32,4 (21,0), *P* < 0,001) and the number of sperm bound to hamster oocytes (1,6 (1,0) versus 2,7 (1,2), *P* < 0,001) were significantly reduced in exposed men [251].

Men (*n* = 12) who were *prenatally* exposed to PCBs and PCDFs during the “Yu-Cheng” incident had a higher percentage of spermatozoids with abnormal morphology (37,5% versus 25,9%, *P* < 0,0001), less motile spermatozoids (35,1% versus 57,1%, *P* = 0,0058), and their spermatozoids were less capable of penetrating hamster oocytes (*P* = 0,017) than 23 unexposed men of similar age [252].

Exposure to dioxin during the Seveso incident led to a decreased sperm quality in men exposed prepubertally, was positively associated with sperm quality in men exposed between ages 10–17, and did not affect sperm quality in men exposed at ages 18–26 [253].

**(iii) PCBs**

Between groups of 34 men with poor semen quality and 31 men with normal sperm quality, Dallinga et al. [254] found no significant differences in organochlorine blood levels. However, among the men with normal semen quality, sperm count and sperm progressive motility were inversely related to the concentration of PCB metabolites [254].

Among 305 young Swedish men 18–21 years old from the general population, Richthoff et al. [255] found weak but statistically significant, negative correlations between PCB-153 levels and both the testosterone : SHBG ratio (*r* = −0.25, *P* < 0.001)—a measure of the biologically active free testosterone fraction—and sperm motility (*r* = −0.13, *P* = 0.02).

Among 212 male partners of subfertile couples who presented to the Massachusetts General Hospital Andrology Laboratory, there were dose-response relationships among PCB-138 and sperm motility (odds ratio per tertile, adjusted for age, abstinence, and smoking, and *P* value for trend were, resp., 1.00, 1.68, 2.35, *P*  value = 0.03) and morphology (1.00, 1.36, 2.53, *P*  value = 0.04). The lack of a consistent relationship among semen parameters and other individual PCB congeners and groupings of congeners may indicate a difference in spermatotoxicity between congeners [256].

Among 195 Swedish fishermen, aged 24–65 years, the subjects in the quintile with the highest PCB 153 concentration (>328 ng/g lipid) tended to have decreased sperm motility compared with the subjects in the lowest quintile (<113 ng/g lipid). The age-adjusted mean difference was 9.9% (95% confidence interval −1.0 to 21%  *P* = 0.08) [257]. No significant associations between p,p′-DDE and semen characteristics or reproductive hormones were found [257].

**(iv) Pesticides**

Fecundity of men was also reported to be decreased in association with occupational exposure to contemporary-use nonpersistent pesticides (see Diamanti-Kandarakis et al. [153]) and even with environmental exposure to such pesticides [258, 259]. Increased odds ratio's for poor semen quality were found (OR (95% CI) in relation to urinary concentrations per g creatinine for several pesticides: alachlor (OR for >0.7 versus <0.15 *μ*g/g: 30.0 (4.3–210)); 2-isopropoxy-4-methyl-pyrimidinol (diazinon metabolite) (OR for >3.0 versus <0.1 *μ*g/g: 16.7 (2.8–98.0)); atrazine (OR for 0.1 versus <0.1 *μ*g/g: 11.3 (1.3–98.9)); 1-naphthol (carbaryl and naphthalene metabolite) (OR for >1.5 versus <1.5 *μ*g/g: 2.7 (0.2–34.2)); 3,5,6-trichloro-2-pyridinol (chlorpyrifos metabolite) (OR for ≥0.5 versus <0.5 *μ*g/g: 6.4 (0.5–86.3) [258]. For increasing 1-naphthol (a metabolite of both carbaryl and naphthalene) tertiles, adjusted odds ratios (ORs) were significantly elevated for below-reference sperm concentration (OR for low, medium, and high tertiles = 1.0, 4.2, 4.2, respectively; *P*-value for trend = 0.01) and percent motile sperm (1.0, 2.5, 2.4; *P*-value for trend = 0.01) [259]. There were suggestive, borderline-significant associations for 3,5,6-trichloro-2-pyridinol (a urinary metabolite of chlorpyrifos and chlorpyrifos-meyhyl) with sperm concentration and motility, whereas sperm morphology was weakly and nonsignificantly associated with both 3,5,6-trichloro-2-pyridinol and 1-naphthol [259].

**(v) Perfluorinated Substances**

Sperm quality was also reported to be diminished in association with more intensive exposure to the perfluorinated substances perfluorooctanoic acid and perfluorooctane sulfonate [260]. Among 105 Danish men from the general population (median age 19 years), men in the highest quartile of combined levels of PFOS and PFOA had a median of 6.2 million normal spermatozoa in their ejaculate in contrast to 15.5 million among men in the lowest quartile (*P* = 0.030) [260].

**(vi) Heavy Metals**

Sperm quality was also reported to be diminished in association with exposure to heavy metals. Among 149 healthy male industrial workers 20–43 years of age residing in Zagreb (Croatia), (98 subjects with slight to moderate occupational exposure to Pb and 51 reference subjects), a Pb-related decrease in sperm density, in counts of total, motile, and viable sperm, in the percentage and count of progressively motile sperm, in parameters of prostate secretory function, and an increase in abnormal sperm head morphology was found. These associations were confirmed by results of multiple regression, which also showed significant (*P* < 0.05) influence of blood cadmium, serum zinc, serum copper, smoking habits, alcohol consumption, or age on certain reproductive parameters. Blood cadmium contributed to a decrease in sperm motility and an increase in abnormal sperm morphology [261].

**(vii) Trihalomethanes**

Higher trihalomethane levels in drinking water might be associated with a decrease in the fecundity of men [262].

**(viii) Air Pollution**

Selevan et al. [263] studied semen quality of young men (18 years of age) living in Teplice, a highly industrialized district with seasonally elevated levels of air pollution, or in Prachatice, a rural district with relatively clean air. Periods of elevated air pollution in Teplice were significantly associated with decrements in several semen measures including proportionately fewer motile sperm, proportionately fewer sperm with normal morphology or normal head shape, and proportionately more sperm with abnormal chromatin [263]. In later research, 36 young men from Teplice were sampled up to seven times over 2 years allowing evaluation of semen quality after periods of exposure to both low and high air pollution. A significant association was found between exposure to periods of high air pollution (at or above the upper limit of US air quality standards) and the percentage of sperm with DNA fragmentation according to sperm chromatin structure assay (SCSA). Other semen measures were not associated with air pollution [264].

**(ix) Phthalates**

Among 234 young Swedish men, subjects within the highest quartile for MEP had fewer motile sperm (mean difference = 8.8%; 95% confidence interval = 0.8–17), more immotile sperms (8.9%; 0.3–18), and lower luteinizing hormone values (0.7 IU/L; 0.1–1.2), but phthalic acid was associated with improved function [265].

DEHP metabolites were measured in spot urine of 463 male partners of subfertile couples who presented for semen analysis to the Massachusetts General Hospital and of men with all semen parameters above WHO reference values. Dose-response relationships were observed for MBP with low sperm concentration (odds ratio per quartile adjusted for age, abstinence time, and smoking status = 1.00, 3.1, 2.5, 3.3; *P* for trend = 0.04) and motility (1.0, 1.5, 1.5, 1.8; *P* for trend = 0.04) [266].

Among 379 men from an infertility clinic, sperm DNA damage was associated with MEP and with MEHP after adjusting for DEHP oxidative metabolites, which may serve as phenotypic markers of DEHP metabolism to “less toxic” metabolites [267].

Swan et al. [268] found that mothers with higher levels of phthalates in their blood during pregnancy had a significantly greater chance of bearing male infants with reduced anogenital distance, a marker for testicular dysgenesis in rodents.

**(x) Anabolic Steroids Used in Food Production**

Residues of anabolic steroids and other xenobiotics used in food production may pose long-term risks for developmental processes in males. For example, in a large study of sperm concentration and fertility in American men by Swan et al. [269], there was a negative association with the number of servings of beef their mothers ate per week while pregnant. The findings of Swan et al. [268, 269] are consistent [270] with the developmental origins of human health and disease (DoHAD) hypothesis [271] and with the testicular dysgenesis syndrome identified by Skakkebaek et al. [188].

**(xi) Endocrine Disruption, Reproductive, and Overall Male Health**

That endocrine disruption has an important impact on the reproductive organs in men is also suggested by considerable evidence indicating that endocrine disruptors contribute to the risk of testicular cancer and prostate cancer (see above) and male urinary tract malformations (see below). Poor semen quality and increased incidence of testis cancer might find their origin in the testicular dysgenesis syndrome [187, 188].

Jensen et al. [272] found significant associations between self-rated health and semen quality and testicular size. Interestingly, good semen quality was associated with a higher life expectancy [273]. The decrease in mortality among men with good semen quality was due to a decrease in a wide range of diseases and was found among men both with and without children; therefore, the decrease in mortality could not be attributed solely to lifestyle and/or social factors. Semen quality may therefore be a fundamental biomarker of overall male health [273].

### 4.6. Development

#### 4.6.1. Male Urinary Tract Malformations

Cryptorchidism and hypospadias are very frequent in Denmark [274–276]. Hypospadias is associated with a slight increase in Follicle Stimulating hormone concentrations indicative of primary testicular dysfunction [275]. Recorded temporal trends, geographical differences, and observations made in wildlife after environmental accidents are compatible with a role of endocrine disrupters in the causation of male urinary tract malformations [274–276]. That endocrine disruption is involved in cryptorchydism and hypospadias is also suggested by the association of cryptorchydism and hypospadias with reduced anogenital distance [277]. 

#### 4.6.2. High Impact of Exposures during Early Life and Certain Time Windows

In utero and during early postnatal life mammalian organisms are much more sensitive than during adult life, not only to mutagenic agents [278], but also to endocrine disruption. Exposures during these periods can entail a high impact, not only on development but also on the risk of disease much later in life. This might be the case for cancer [279–283], obesity [284–286], the metabolic syndrome [286], diabetes [286], cardiovascular disease [286], neurodegenerative disease such as Alzheimer and Parkinson [287–294], and mental retardation [295]. Prenatal exposures might even contribute to psychoses [296, 297]. Especially during certain time windows, when critical proliferation, differentiation, or migration processes take place, a highly increased sensitivity to disrupting agents must be taken into account, especially when these agents are receptor binding [271]. This insight led to the new paradigm on “developmental origins of human health and disease” [271].

Early life exposure (late embryonic and/or early postnatal) to low doses of PCBs [298–300] or soy [301] significantly and adversely affected mating behaviors in female rats. Early postnatal treatment with coumestrol (a phytoestrogen) diminished masculine and feminine sexual behaviors [302, 303]. These results are consistent with an impact of EDCs on the neuroendocrine hypothalamus (see also Section 5.1.10).

Internal prenatal exposure to perfluorooctanoic acid (PFOA) was inversely associated with birth weight in some studies, but not in others [226].

#### 4.6.3. Males Often More Sensitive Than Females

In mammalians, the ontogenetic evolution towards the female phenotype appears in some way to be the default process and occurs to a large extent without sex hormone influence [304]. The ontogenetic evolution towards the male phenotype on the contrary occurs under the influence of sex hormones produced by the testis (both testosterone and estradiol are necessary for normal sexual differentiation of the male brain) [304] and so probably is more vulnerable to endocrine disruption. Also, sexual differentiation of the female and male brain differs due in part to alfa-fetoprotein, which protects the brain from effects of maternal estrogens. Alfa-fetoprotein knockout mouse females are masculinised and defeminised in brain and behaviour, providing further support for a role of estrogens in the masculinisation of the brain. Additional differences in brain development between the sexes may result from even subtle differences in the timing of hormone exposures, as the mammalian brain is exquisitely sensitive to hormones in late embryonic and early postnatal time periods, and even small differences may exert large effects [304]. There has been considerable and consistent research that shows that PCBs, phytoestrogens, fungicides, pesticides, and other xenobiotics can disrupt brain sexual differentiation [304].

Even adult males and females do not necessarily react in the same way to exogenous substances. In the Flemish biomonitoring, changes in gene expression associated with internal exposure to DDE, hexachlorobenzene, marker PCBs and dioxin-like activity were predominantly in opposite direction for men and women [305].

## 5. Mechanistic Considerations

Endocrine-disrupting agents can act through many different mechanisms. This is best documented for agents interfering with sex hormones, especially xeno-estrogens, and most of the evidence below stems from this field. 

### 5.1. Mechanisms Leading to Endocrine Disruption

#### 5.1.1. Activation of the Classical Nuclear Receptors

In the classic view, estrogens control gene networks and modulate target cell activities through activation of the ER*α* and ER*β* nuclear receptors, which bind estrogen responsive elements (EREs) in the promoters of target genes and regulate target gene expression [306]. 

Initially, due to the often limited effect of interaction with ERs (e.g., BPA is, in this respect, 1000–2000 fold less potent than 17*β*-estradiol), it was thought that the significance of xenoestrogens was low [307, 308]. However, the last decade(s), a number of other mechanisms were discovered, often triggered at low concentrations of xenoestrogen. This will be further discussed in this section. 

Endocrine disruption of the thyroid system too can occur through interference with thyroid hormone receptor-dependent transactivation [126]. 

#### 5.1.2. Effects of Activation of Receptors Differs in Function of the Ligand

After activation by binding to a ligand resulting in a conformation change, receptors can act as transcription factors, interacting with coactivators and corepressors and with DNA sequences [309, 310]. However, the change in conformation differs in function of the ligand [311–314]. Differences in conformation can be expected to be associated with differences in function and thus differences in regulatory activity on gene expression. That is what has been observed: receptors bound to xenobiotic ligands do not have exactly the same influence on gene expression as receptors bound by endogenous ligands [315, 316]. This has been studied for several xenoestrogens, such as octylphenol, nonylphenol, endosulfan, and kepone by Wu et al. [317], who demonstrated the highly structure-dependent induction of luciferase activity in MCF-7 and MDA-MB-231 breast cancer cells transfected with a construct linked to ER*α* and luciferase. 17Beta-estradiol and the phytoestrogen coumestrol even had opposite effects on the regulation of estrogen receptor beta mRNA in the brain of rats [318].

#### 5.1.3. Activation of Membrane-Bound Estrogen Receptors: mER*α*, mER*β*, and GPR30

Until recently, most studies have focused on the slow, genomic phase of steroid responses based on activation of nuclear receptors and acting through modifications of transcription and protein synthesis. However, steroid hormones can also induce rapid (seconds to minutes) nongenomic responses based on plasma membrane receptors and acting through second messenger-triggered signal cascades [319].

In the past, the basis for calling an estrogen “weak” or “strong” has been entirely dependent upon the nuclear transcription signalling mechanism [320]. It is now becoming clear that “weak” activity via one pathway does not necessarily predict the potency of a hormone or mimetic acting via another signalling pathway. Though the activities of most environmental estrogens have been called “weak” for many years because of their inability to initiate nuclear retention and transcriptional effects, we now see that they are quite potent initiators of signal cascades emanating from the membrane [319]. 

Two types of membrane estrogen receptors, mER*α* and mER*β*, are likely the same proteins as the nuclear receptors ER*α* and ER*β*, transported to the plasma membrane by yet undetermined mechanisms [306]. As to nongenomic actions of activated mERs, one example is Ca^++^ release that can lead to changes in cell motility, intra- and extracellular signalling processes, and rapid hormone secretion (including prolactin) trough exocytosis. Changes in prolactin (PRL) secretion are associated with hormonal regulation of lactation, cell proliferation, the cellular immune response, and parental/maternal behaviour [321]. Xenoestrogens such as dieldrin, endosulfan, o,p′-DDE, nonylphenol, bisphenol A, coumestrol, and diethylstilbestrol are known to affect Ca^++^ influx and PRL release. Interestingly, Wozniak et al. [321] found these xenoestrogens to affect one or more of the above pathways in a specific way. Differences between xenoestrogens included the concentration range (pM or nM) in which they were active, as well as in the temporal pattern in which they induced a specific mechanism, for example, early- or late-phase activation or an early and sustained activation. This illustrates the complexity of xenoestrogen induced endocrine disruption.

Another, relatively recent, discovery is the seven-transmembrane estrogen receptor, GPR30, that activates alternative estrogen signalling. High binding affinities for GPR30 were demonstrated in ER-negative cells for bisphenol A, genistein, zealonone, and nonylphenol. Lower binding affinities were found for Kepone, p,p′-DDT, o,p′-DDE, and 2,2,5-trichloro-4-biphenylol (2,2,5,-PCB-4-OH). GPR30 is expressed in a broad range of tissues, such as the brain, placenta, ovaries, testes, prostate, heart, pancreas, lungs, skeletal muscle, colon, vascular epithelial, and lymphoid tissues. Thus, xenoestrogens could mimic estrogen action in all of these tissues and inappropriately activate estrogen signalling [322].

The rapid “nongenomic” mechanisms have been shown to interact with cytoplasm signal transduction molecules such as cAMP and adenylate cyclase, calcium, PI3K, PKB, Src (and consequent activation of kinases Erk1 and Erk2 in the Src/Ras/Erk-cascade) and G-proteins or directly with secondary transcription factors such as AP-1 (activator protein 1), STATS (signal transducer and activator of transcription), NF*κ*B (nuclear factor kappa-light-chain-enhancer of activated B cells), and Sp1 (specificity protein 1) [323, 324].

#### 5.1.4. Cytoplasmic Interactions

Besides with nuclear- and plasmamembrane-associated ER receptors, estrogens can also interact with targets within the cytosol. One of the best studied examples is the activation of Src/Ras/ERK (MAPK) pathway, in a mechanism linked to the proliferative effects of estrogens. Binding of estrogens to cytosolic ER receptors determines the interaction of ERs with Src, changing the conformation of the kinase to an active state and leading to the activation of the Src/Ras/ERK signaling cascade [325]. Another example is the modulation of nitric oxide (NO). Estrogen activates endothelial NO synthase (eNOS, the enzyme responsible for NO production). This activation in endothelial cells by ER*α* involves phosphatidylinositol 3-kinase (PI3K) and an ER*α*-eNOS signalling complex in the endothelial caveolae. Binding of estrogen to the ER*α* will, via a direct physical interaction with PI3K, activate serine/threonine protein kinase B (Akt), which in turn will phosphorylate and activate NOS [325].

#### 5.1.5. Cross-Talk between Genomic and Nongenomic Pathways

As can be deduced from previous chapters, xenoestrogens can influence several mechanisms simultaneously. Therefore, Silva et al. [323] investigated the differential action of xenoestrogens, acting on both nuclear (genomic) as well as on extranuclear (non-genomic) pathways, and possible cross-talk between these two mechanisms triggered by the xenoestrogenic chemicals o,p′-DDT, p,p′-DDE, and *β*-HCH in MCF-7 cells. Therefore, expression of estrogen responsive genes and phosphorylation of Src, Erk1, and Erk2 were measured after exposure to these estrogenic compounds. The researchers found strong similarity between E2, o,p′-DDT, and *β*-HCH in gene expression and phosphorylation patterns, as well as in cell proliferation. This is despite of the lack of affinity of *β*-HCH for the ER binding domain. p,p′-DDE, however, influenced estrogen-related gene expression, but did not phosphorylate Src and Erk1/Erk2. Other authors have also found diversity of effects between different xenoestrogens, such as bisphenol A (unable to induce Erk activation) and endosulfan and p-nonylphenol (rapidly inducing Erk1/Erk2 phosphorylation) [326]. 

Li et al. [327] demonstrated that the activation or inhibition of kinases (including ERk1/Erk2, PI3K, PKC, PKA) by xenoestrogens (bisphenol-A, nonylphenol, octylphenol, endosulfan, kepone, 2,2-bis(p-hydroxyphenyl)-1,1,1-trichloroethane [HPTE], and 2′,3′,4′,5′-tetrachloro-4-biphenylol) depends on chemical structure. These studies have shown that estrogenic compounds can both induce rapid nongenomic and genomic responses, or both. These differences could (in part) be explained by the existence of both the classic ER binding domain, as well as an “alternative binding pocket”(inducing signaling cascades), each binding-specific (xeno)estrogens [323]. Silva et al. [323] conclude that both genomic and non-genomic effects and crosstalk should be brought into consideration when screening for environmental estrogens.

#### 5.1.6. Activation of Estrogen-Related Receptors

Estrogen related receptors (ERR) are a subfamily of orphan nuclear receptors closely related to ER*α* and ER*β*. Three of these ERR are known: ERR*α*, ERR*β*, and ERR*γ*. ER and ERR show a considerable amount of similarity in aminoacid sequence, but E2 does not bind ERRs. However, ERR can bind to estrogen response elements, which suggests a possible overlap between ER and ERR action [328]. 

ERRs show spontaneous transcriptional activity, which is known to be repressed by a few chemicals. For example, DES represses the molecular activities of ERRs, however, to a considerably lesser extent than its action as an ER activator. Another inverse agonist of ERR*γ* is 4-hydroxytamoxifen (4-OHT). On the other hand, bisphenol-A has been observed to have a distinct antagonist action to the inverse agonist activity of 4-OHT, thus preserving ERR-activity in the presence of 4-OHT [328].

#### 5.1.7. Cross-Talk with Estrogen Receptors after Binding on Other Receptors

Although their antiestrogenic actions are well described, dioxins can also induce endometriosis and estrogen-dependent tumours, implying possible oestrogenic effects. A heterodimer of the dioxin receptor (AhR) and Arnt, which are basic helix-loop-helix/PAS-family transcription factors, mediates most of the toxic effects of dioxins. Ohtake et al. [329] showed that the agonist-activated AhR/Arnt heterodimer directly associates with estrogen receptors ER-alpha and ER-beta. This association resulted in the recruitment of unliganded ER and the coactivator p300 to estrogen-responsive gene promoters, leading to activation of transcription and estrogenic effects. The function of liganded ER was attenuated. This mechanism is compatible with as well oestrogenic as antiestrogenic effects. Ohura et al. [330] demonstrated that AhR-induced activation of ER is dependent on ligand structure and does not necessarily occur for every AhR ligand.

#### 5.1.8. Changes in DNA Methylation or Histone-Modifications

Epigenetic changes, such as DNA methylation and histone modifications, have been shown to be involved in the mechanisms related to endocrine disruption. Developmental exposure to estradiol and bisphenol A increased susceptibility to prostate carcinogenesis and regulated phosphodiesterase type 4 variant 4 expression epigenetically, through changes in DNA methylation [331]. Transient exposure of a gestating female rat during the period of gonadal sex determination to the endocrine disruptors vinclozolin (an antiandrogenic compound) or methoxychlor (an estrogenic compound) induced an adult phenotype in the F1 generation of decreased spermatogenic capacity (cell number and viability) and increased incidence of male infertility. These effects were transferred through the male germ line to nearly all males of all subsequent generations examined (i.e., F1 to F4). The effects on reproduction correlate with altered DNA methylation patterns in the germ line [332, 333]. Early life exposures to EDCs may alter gene expression in hypothalamic nuclei via nongenomic, epigenetic mechanisms, including DNA methylation and histone acetylation [304].

#### 5.1.9. Genomic Instability by Interfering with the Spindle Figure

Reports show that estrogens, including E2, E3, and bisphenol A, induce micronuclei in MCF-7 cells, indicating that the (xeno) hormones have the ability to cause genomic instability [334]. Kabil et al. [335] observed that ER antagonists (interfering with the transcriptional activity of the ER and blocking promotion of ER-dependent gene expression) did not prevent micronucleus formation by these estrogens. On the other hand, coadministration of estrogens and kinase inhibitors, interfering with the extracellular signal triggered Scr/Raf/Erk signalling pathway (see Section 5.1.3), led to significantly less micronucleus formation. Kabil et al. [335] suggest that estrogens induce micronuclei formation by enhanced Scr/Raf/Erk stimulation, disturbing the localisation of Aurora B kinase to kinetochores and leading to improper chromosome segregation. 

#### 5.1.10. Interference with Hormonal Feedback Regulation and Neuroendocrine Cells

Along with the direct influence of EDCs on estrogen or androgen actions, they can affect endogenous steroid production through negative and positive feedback, effects that may differ depending on developmental stage [153]. Neuroendocrine systems function as links between the brain and peripheral endocrine systems and are responsible for the control of homeostatic processes including reproduction, growth, metabolism, energy balance, and stress response. As stated by Gore [336], disruption of neuroendocrine homeostasis by endocrine-disrupting chemicals can lead to a series of perturbations. 

GnRH (Gonadotropin-releasing hormone) neurons in the hypothalamus control reproductive function in vertebrates. GnRH binds to its receptors on cells named gonadotropes, which synthesise and release luteinizing hormone (LH) and follicle stimulating hormone (FSH). These hormones then bind to receptors on ovary and testes to cause steroidogenesis and gametogenesis. Disturbance of GnRH levels can be induced by amongst others PCBs, o,p′-DDT, bisphenol A and is dependent on the chemical and on timing of treatment [336]. 

Evidence exists for neuroendocrine disruption of the hypothalamic-pituitary-thyroid system, with implications on (amongst others) metabolism and energy balance. For example, PCBs reduce the thyroxin and TSH (thyroid stimulating hormone) response to TRH (thyrotropin releasing hormone), which indicates hypothalamic and/or pituitary deregulation. Thyroid disruption also has consequences for neural development [336]. Disturbance of neuropsychic development in association with internal exposure to PCBs has indeed been observed in biomonitoring studies on adolescents [337] as well as on 36-month-old children, in this last instance in association with PCB levels measured in cord blood (unpublished results from the Flemish biomonitoring, Vermeir et al. in preparation). 

Bisphenol A is another endocrine disruptor identified as a developmental thyroid toxicant [336]. Furthermore, the obesogenicity of DES has been supposed to involve action on the developing hypothalamic circuits, which are important for the energy balance.

Ceccarelli et al. [338] have shown in rats that exposure during early puberty to the estrogenic chemicals 17-ethinylestradiol (EE) and bisphenol-A (BPA) has a distinctive effect on ER-*α*-expressing neurons, in key brain areas involved in reproductive behavior. The number of ER-*α* containing cells and the testosterone and estradiol serum levels were modified, and the changes were both sex-dependent and different in the short and long term.

Also, exposure to hormonally active substances such as exogenous endocrine-disrupting chemicals (EDCs), may result in improper hypothalamic programming, thereby decreasing reproductive success in adulthood [304]. Furthermore, transmission of neuroendocrine effects to future generations has also been observed for vinclozolin with significant alterations in brain gene expression and behavior in F3 descendants [339].

#### 5.1.11. Effects on the Metabolism of Hormones

Xenoestrogens have been shown to affect steroidogenic enzymes, including 3b-HSDs and 17b-HSDs (hydroxysteroid dehydrogenases), aromatase, sulphatases, and sulphotransferases. Mostly, steroidogenesis is inhibited by xenoestrogens [123]. 

Atrazine was observed to stimulate aromatase activity in some cell types, which leads to a higher synthesis of estradiol [340]. On the other hand, DDT and several metabolites, lindane, MEHP (phthalate metabolite), and several organotins were reported to inhibit aromatase activity in some cell types [123]. 

Hydroxylated metabolites of polyhalogenated aromatic hydrocarbons showed potent inhibition of estrogen sulfotransferase [341], an enzyme active in the excretion of estrogens, which elevates bioavailable estrogens in target organs. 

Disruption of thyroid function occurs mainly through prereceptor regulation of ligand concentration [136, 137]. Organohalogens are among the chemicals that can induce a decrease in circulating thyroid hormone levels [137]. This is through at least three distinct mechanisms. First, the chemical can directly affect the thyroid gland to decrease the synthesis of TH [342]. Second, reduced TH levels can be caused by enhanced biliary excretion of T4 due to the induction of UDP-glucuronyltransferases [343]. Third, the chemical may displace binding of the natural ligand, T4 to TTR, which could result in deiodinase inactivation and biliary excretion of the hormone [344, 345]. Although all three mechanisms might be responsible for the decreases in circulating TH by organohalogens (parent compound and metabolites), high affinity binding of organohalogens to TTR might be the key factor in eliciting the biological effects [137].

#### 5.1.12. Effects on Oxidative Metabolism through Activation of the Pregnane X Receptor

The expression of many genes involved in xenobiotic/drug metabolism and transport is regulated by at least three nuclear receptors or xenosensors: aryl hydrocarbon receptor (AhR), constitutive androstane receptor (CAR), and pregnane X receptor (PXR). These receptors establish crosstalk with other nuclear receptors or transcription factors controlling signalling pathways that are important to the homeostasis [346]. The pregnane X receptor (PXR) is activated by many drugs and environmental pollutants. A significant proportion (54%) of compounds with estrogenic activity or those able to bind ER were found to be hPXR activators. This was the case for classical estrogens such as estradiol and ethynylestradiol, for some antiestrogens such as 4-hydroxytamoxifen, for some mycoestrogens, for bis (2-ethylhexyl) phthalate, dibutylphthalate, and benzyl butyl phthalate, for several alkylphenols, for some UV-screens, and (but only weakly) for the fragrance galaxolide [31]. Also perfluorooctanoic acid (PFOA) was shown to activate the constitutive androstane receptor and pregnane X receptor [226]. The constitutive androstane receptor could also play a similar role but binds much less chemicals than the PXR receptor [347]. Differences in the pregnane X receptor are thought to underlie differences in xenobiotic metabolism between species [31]. 

The pregnane X receptor, which regulates several cytochrome P450 enzymes crucial in the oxidative metabolism of a wide range of chemicals, can thus play a role as well in the inactivation of toxic or carcinogenic chemicals as in the activation of procarcinogens and could also act as a protector of the endocrine system from chemical perturbation [31]. 

### 5.2. Low-Dose Effects and Nonlinear, Biphasic, and Nonmonotonic Dose: Effect Relationships

#### 5.2.1. Experimental Data

For receptor binding xenohormones, it can be expected that even extremely low doses have some effect. Indeed, dose response curves for hormonal effects can be expected and often do follow Michaelis-Menten kinetics [348] implying a supralinear dose-response relationship in plots featuring linear scales for both response in ordinate and exposure in abcis. 

Biological and health effects of pollutants capable of binding to receptors show complex dose-response relationships including nonmonotonic associations [54, 349–352].

Furthermore, using an ordinary differential equation-based computational model, Li et al. [353] demonstrated that nonmonotonic dose-responses in gene expression can arise for exogenous ligands of steroid hormone receptors in an endogenous hormonal background. 

In the case of some endocrine disruptors such as bisphenol A, exhibiting other modes of endocrine disruption in addition to binding to nuclear estrogen receptors, such as alterations in the synthesis or the metabolism of endogenous hormone and binding to plasma membrane estrogen receptors and also to androgen and thyroid hormone receptors, nontraditional dose response curves such as inverted U or U-shaped curves were observed [36]. As stated by the Chapel Hill bisphenol A expert panel [36], below the concentration range in which “pharmaceutical” effects are detected by classical toxicology, some substances, such as BPA, show biological effects due to disruption of cellular signalling mechanisms. For these substances, the safe level determined by classical toxicology does not protect against effects in the cell signal disrupting range [36]. 

While BPA was initially considered to be a “weak” estrogen based on a lower affinity for estrogen receptor alpha relative to estradiol [354, 355], research shows that BPA is equipotent with estradiol in its ability to activate responses via recently discovered estrogen receptors associated with the cell membrane [321, 356–358]. It is through these receptors that BPA stimulates rapid physiological responses at low picogram per ml (parts per trillion) concentrations. Also, Lemmen et al. [354] found BPA to be more potent in activating embryonic ERs than would be expected on the basis of its in vitro activity.

That dose-effect relationships can be very complex for endocrine disruptors is evident from following examples: BPA has been found to have a greater effect on prostate tumor cell proliferation at 1 nanomole concentrations than it does at 100 nanomoles [359]. The plastic softener DEHP has been found to upregulate aromatase expression in neonatal male rats at doses above the LOAEL, but downregulate it at doses far below the LOAEL [360]. In an extreme example of different high-dose and low-dose effects, arsenic causes multiple organ failure at high doses, suppresses glucocorticoid hormone induction at low levels, yet enhances the same process at lower levels still [361]. 

#### 5.2.2. Low-Dose Effects in Humans

For more than two decades, the hypothesis of endocrine disruption has become a contested area of science. Some scientists have argued that isolated incidents of high levels of chemical contamination have affected health and reproduction of organisms, but that generally the levels of contamination of human beings have been far too low to have had effects. In the late 1990s, the U.S. National Academy of Sciences [362] set up a committee to examine the evidence on endocrine disruptors. The Academy considered that xeno-estrogens were at least a thousand times less powerful than endogenous hormones and that the levels encountered by humans were low and so were unlikely to yield any serious effects on human health. However, several of the observations mentioned under 4.5.2. and under 4.6. indicate that environmental exposures to very low doses do indeed have effects on humans. The observations made in the Flemish biomonitoring show that very low dose internal exposures to cadmium and to organochlorine pollutants (exposures under the median level of exposure in Flemish adolescents) are associated with detectable and sometimes pronounced differences in serum sex hormone levels [363], in sexual maturation [364], in gynaecomastia in boys [364], in height and in body mass index [217]. For several parameters, the exposure-effect relation was more pronounced per unit of dose at internal exposures under the median than above the median [217, 363]. Premature thelarche has been reported in girls exposed to phthalates [365]. In the Flemish biomonitoring campaign 2007–2011, we observed that internal exposures actually occurring in adolescents aged 14-15, having no occupational exposure, to arsenic, polycyclic aromatic hydrocarbons, phthalates, organophosphorus insecticides, perfluorooctanoic acid, and the musk galaxolide, showed associations with serum hormone levels, with sexual maturation or with damage to DNA in peripheral blood cells (unpublished results, see http://www.milieu-en-gezondheid.be/resultaten/2007-2011/studiedag%2021-12-2011/abstract%20NVL.pdf).

### 5.3. Effects of Combined Exposures

The large number of endocrine-disrupting compounds present in the environment raises questions about the effects of simultaneous exposure to multiple compounds. However, relatively few studies have discussed combination effects.

Recently, Correia et al. [366] have tested combination effects of the (xeno)estrogens E_2_, EE_2_, and bisphenol A in fish, using vitellogenin induction as an endpoint. Effects of combined exposure to several (xeno)estrogens at equipotent concentrations were in high agreement with the predictions made with the model of concentration addition (based on the assumption that chemicals act via a similar mechanism to elicit an effect, such that one chemical acts as a dilution of the other and can be substituted at a constant proportion for the other), which points to additive action of these compounds. Zhang et al. [367] have also assessed the effect combinations of (xeno)estrogens (E2, EE2, bisphenol A and 4-tert-octylphenol) and observed that the concentration addition model agreed best with the observed combination effects, confirming additive effects of these substances on vitellogenin induction.

Other endpoints, however, show far more complex outcomes after combined exposure. Kochukov et al. [368] have previously demonstrated peaks of ERK-phosphorylation after E2, ethylphenol (EP), octylphenol (OP), propylphenol (PP), nonylphenol (NP), and bisphenol-A (BPA) exposure in pituitary cells. These peaks occurred at 2.5–5 minutes, 10–30 minutes, and 60 minutes for E1, E2, and BPA (3-peak oscillation), while for the alkylphenols and E3 a two-peak oscillation was observed with oscillations at 2.5–5 minutes and at 60 minutes (missing the intermediate peak). Later Jeng and Watson [369] studied the effects of combined exposure of these xenoestrogens with E1, E2, or E3. For cotreatment with alkylphenols, the first peak of the physiological estrogens (2.5–5 minutes) was abolished or blunted, while the second peak (10–30 minutes) was augmented. The 3rd peak, however, was mostly declined, often far below the response of the individual (xeno)estrogens. Thus, a temporal pattern with both synergistic (second peak) and antagonistic effects (3rd peak) was observed, which resulted in a transformation of the response from a three-peak oscillation to a single intermediate peak. The strength of the individual xeno-estrogen tended to be predictive for their ability to inhibit actions of the natural estrogens when combined. BPA had an even more complex response, highly dependent on the dose. At very low concentrations (10^−14^ M), combinations of physiological estrogens with bisphenol-A caused a response similar to alkylphenols (synergistic effect at the second peak, antagonistic effect at the third peak). However, at 10^−9^ M concentrations, a synergistic effect was observed at both the second and third peaks [369]. Jeng and Watson [369] also studied dose dependency of effects at the 5-minute time peak. Most alkylphenols had a higher effect on ERK activation at higher doses, except for OP which showed more activity at lower doses. In combination, alkylphenols generally enhanced the effect of physiological estrogens at lower concentration but severely disrupted them at higher concentrations. BPA alone showed a non-monotonous response, with higher effects at low (10^−15^ M) and high (10^−7^ M) concentrations, and lower effects at intermediate concentrations. Remarkably, in combination, BPA was most disruptive of the physiological estrogen at the low (10^−15^ M) and high (10^−7^ M) concentrations, while physiological estrogen effects were unaffected or enhanced at intermediate concentrations. In other words, in concentration ranges where BPA had highest estrogenic activity on its own, it also had the highest inhibitory effect on physiological estrogens when combined [369].

Zsarnovszky et al. [358] have made similar observations with BPA and E2 in cerebellar neurons, with BPA induced increases of pERK positive cells at low (10^−10^–10^−12^ M) and high (10^−7^–10^−6^ M) concentrations, but BPA did not affect basal ERK signaling at intermediate concentrations, while coadministration of E2 (10^−10^ M) and BPA (10^−12^–10^−10^ M) inhibited ERK activation. Thus, BPA can both mimic or block some actions of E2. This behavior could be explained by the existence of an additional high affinity BPA-binding site with inhibitory activity, which becomes available only upon ligand binding at the high-affinity site of the rapid ERK-stimulating receptor [358].

The observation that pharmaceutical (fadrozole) and environmental aromatase inhibitors (tributyltin) do not block the effect of the xenoestrogen dichlorodiphenyltrichloroethane (o,p-DDT) suggests that in the environment, exposure to seemingly antagonistic EDCs does not necessarily lessen the harmful impacts of these compounds [370].

These observations illustrate the complexity of combined exposures to multiple endocrine disrupting compounds (and physiological estrogens), as well as the risks of endocrine disruption, even at very low doses of exposure. Furthermore, it is important to remember that a lot of different parameters, such as the coexistence of multiple ER isoforms, influence of homo- and heterodimers, posttranslational modifications of the receptors, localization at the membrane, and the changeable nature of the receptor signaling complex all likely contribute to the complex properties of (xeno)estrogens [358].

## 6. Conclusion

There is substantial evidence indicating that endocrine disruptors contribute to the risk of cancer, developmental problems, diabetes, and possibly also obesity and the metabolic syndrome. Also, it seems highly likely that endocrine disruptors can contribute to infertility and subfertility. That is why both the Endocrine Society [153] and the American Chemical Society [371] (with 161.000 chemical scientists and engineers as members, the world's largest scientific society) recently issued scientific statements on endocrine disruption. In their statements, these scientific societies recommend increased efforts at identifying and studying endocrine disruptors, expansion of education throughout the formal education structures in universities and schools, and better information to the public in general and to health care professionals and to chemists in particular. The Endocrine Society stresses the importance of the precautionary principle in the absence of direct information regarding cause and effect and considers the principle to be critical to enhancing reproductive and endocrine health. The American Chemical Society recommends more Green Chemistry research aimed at identifying and developing functional alternatives that do not have endocrine-disrupting activity. It remains, however, very difficult to determine which substances, at which point in time and at which concentrations, actually increase risk. Implementing the physical-chemical hygiene is in this context certainly indicated.

## Figures and Tables

**Table 1:** Some xenoestrogens, xenoandrogens, antiestrogens, and antiandrogens.

Substance	Some data on occurrence, use^a^	References mentioning xenooestrogenic activity	References mentioning xenoandrogenic activity	References mentioning antiestrogenic activity	References mentioning antiandrogenic activity
Naturally occurring substances					

20S-Protopanaxadiol	Gastrointestinal metabolic product of ginsenosides			[20]	
Alpha-zearalenol	Metabolite of zearalenone, used as growth promoter in beef cattle in certain non-European Union countries	[21]			
Apigenin	Flavonoid phytoestrogen	[22]			
Ferutinine	Sesquiterpenoid, in plants of Umbelliferae family	[23]			
Formononetin	Isoflavonoid, in red clover and soy extracts	[24]			
Genistein	Isoflavonoid, in red clover and soy extracts	[24]		[25]	
Kaempferol	Flavonoid phytoestrogen	[22]			
Methoxylated brominated diphenyl ethers	Occur naturally in marine organisms			[26]	[26]
Zearalenone	Mycotoxin, food contaminant	[21]			

Industrial products and pollutants including organohalogens, plastic components, detergents					

3,9-Dihydroxy-benz[a]anthracene	Potential metabolite of benz[a]anthracene (PAH, mainly < incomplete combustion)	[27]			
3-Methylcholanthrene	PAH, mainly < incomplete combustion	[28]		[29]	
4-n-Octylphenol	Intermediate in production of and degradation product of octylphenol ethoxylates used to in rubber, in pesticides and paints	[22, 30, 31]			
4-Nonylphenol	Precursor to and degradation product of commercially important detergents	[30]			
4-n-Propylphenol	Degradation product of surfactants	[31]			
4-Tert-butyl 2-methylphenol	Industrial product and environmental contaminant	[22]			
4-Tert-octylphenol	Used in the manufacture of nonionic surfactants	[22, 31, 32]			
7,12-Dimethyl-benz(a)anthracene	PAH, mainly < incomplete combustion	[33]			
Benzotriazole	Anticorrosive agent used in aircraft de-icing, antifreeze fluids, and dishwasher detergents			[34]	
Benzyl butyl phthalate	Plasticizer for polyvinyl chloride in vinyl floor tiles, vinyl foam, and carpet backing, in cellulosic resins	[35]			
Bisphenol A	Used in the production of polycarbonate plastic and epoxy resins used as coatings on the inside of food and beverage cans	[22, 36, 37]			
Dibutylphthalate	Mainly used as plasticiser, also in paints, inks, and cosmetics	[35]			
Dichlorostyrene	Intermediate in chemical industry			[38]	
Dodecylphenol	Used in phenol resins in adhesives and paints	[31, 39]			
Nonylphenols	Precursor to and degradation product of commercially important detergents	[22, 31, 32]		[40]	
Polybrominated biphenyl ethers (PBDEs)	Flame retardants used in plastics, foams, building materials, electronics, furnishings, motor vehicles	[26, 41, 42]		[43, 44]	
Polychlorinated biphenyls (PCBs) (some congeners)	Industrial products, used as dielectric and coolant fluids	[45–50]	[51]	[52, 53]	[54]

Substances used in personal care products					

3-Benzylidene camphor	UV filters, used in sunscreens and cosmetics	[55]		[55]	[55]
4,4-Dihydroxy-benzophenone	UV filter, used in sunscreens and cosmetics	[55]			[55]

4-Hydroxybenzoic acid n-butyl ester, 4-hydroxybenzoic acid ethyl ester, 4-hydroxybenzoic acid propyl ester	Preservatives in foods, drugs, and personal products	[56]			

4-Hydroxybenzophenone	Metabolite of UV filter, used in sunscreens and cosmetics	[55]			[55]
4-Methylbenzylidene camphor	UV filters, used in sunscreens and cosmetics			[55]	[55]
Benzophenone-1	UV filter, used in sunscreens and cosmetics	[55]			[55]
Benzophenone-2	UV filter, used in sunscreens and cosmetics	[55, 57]	[55]		[55]
Benzophenone-3	UV filter, used in sunscreens and cosmetics	[55]		[55]	[55]
Benzophenone-4	UV filter, used in sunscreens and cosmetics	[55]		[55, 58]	[55]
Benzyl salicylate	UV filter, used in sunscreens and cosmetics	[55]		[55]	[55]
Ethoxylated ethyl 4-amino benzoate	UV filter, used in sunscreens and cosmetics			[55]	
Ethyl-4-aminobenzoate	UV filter, used in sunscreens and cosmetics	[55]			[55]

Eusolex 2292, Eusolex 6300, Eusolex 6007, Eusolex HMS	UV filter, used in sunscreens and cosmetics	[56, 59]			

Galaxolide (HHCB)	Fragrance, used in sunscreens and cosmetics	[56]		[60]	[60]
Homosalate	UV filter, used in sunscreens and cosmetics		[55]	[55]	[55]
Isopentyl-4-methoxycinnamate	UV filter, used in sunscreens and cosmetics		[55]	[55]	[55]
Octocrylene	UV filter, used in sunscreens and cosmetics		[55]	[55]	[55]
Octyl dimethyl para amino benzoate	UV filter, used in sunscreens and cosmetics			[55]	[55]
Octyl salicylate	UV filter, used in sunscreens and cosmetics		[55]	[55]	[55]
Octyl-methoxycinnamate	UV filters, used in sunscreens and cosmetics		[55]	[55]	[55]
Para amino-benzoic acid	UV filter, used in sunscreens and cosmetics			[55]	
Phenyl salicylate	UV filters, used in sunscreens and cosmetics	[55]		[55]	[60]
Tonalide (AHTN)				[60]	[60]

Pesticides					

2,4,5-Trichloro-phenoxyacetic acid	Herbicide, defoliant used in Agent Orange in Vietnam			[61]	
3-(2,2-Dichlorovinyl)-2,2-dimethylcyclopropene carboxylic acid	Pyrethroid insecticide metabolite			[62]	
3-Phenoxybenzoic acid	Pyrethroid insecticide metabolite			[62]	
Alachlor	Acid amide herbicide against grasses and broadleaf weeds. Use as herbicide banned in European Union (2006)			[63]	
Aldrin,	Organochlorine insecticide, widely used until 1970s	[63]		[61]	[64]
Atrazine	Still widely used triazine herbicide, banned in the European Union	[65]			
Azinphos-methyl	Organophosphate insecticide, banned in the European Union (2006)				[66]
Benomyl	Carbamate fungicide has been used on fruits	[61]			
Bromopropylate	Carbinol acaricide, used on fruits and legumes, in viticulture	[63]			[63, 66]
Carbaryl	Carbamate insecticide, widely used in gardens, agriculture, and forestry			[61]	
Chlomethoxyfen	Diphenyl ether herbicide, used on rice				[63]
Chlordane,	Organochlorine pesticide, banned in USA in 1988	[61, 63]			[63, 64]
Chlordecone	Organochlorine insecticide and fungicide, banned by the Stockholm Convention on persistent organic pollutants	[22, 61]		[61]	
Chlornitrofen	Diphenyl ether herbicide				[63]
Chlorpropham	Carbamate herbicide and plant growth regulator				[66]
Chlorpyriphos	Organophosphorus insecticide, highly used on corn and fruit trees, still frequently used in Danish greenhouses in 2001	[63, 67, 68]			
Cycloprothrin	Pyrethroid insecticide used in agriculture			[62]	
Cyfluthrin	Pyrethroid insecticide, agriculture, home, garden	[63]		[62]	[63]
Cyhalothrin	pyrethroid insecticide, agriculture, home, garden			[63]	[66]
Cypermethrin,	Pyrethroid insecticide, in agriculture, home, garden	[63, 69]			
Cyprodinil	Fungicide used in agriculture and on fruit				[66]
DDE	DDT metabolite				[63, 66]
DDT,	Organochlorine pesticide. Banned	[22, 61, 63]			[63, 64]
Deltamethrin	Pyrethroid insecticide, widely used, still frequently used in Danish greenhouses in 2001	[67]		[63]	
Dichlorvos	Organophosphate insecticide widely used to control household pests, also on outdoor fruit, and vegetable crops. Frequently used in Danish greenhouses in 2001				[67]
Dicofol	Organochlorine pesticide for mite control on agricultural crops, ornamentals, and around buildings	[63]			[63, 66]
Dieldrin	Organochlorine pesticide, now banned in many countries	[61, 63, 67]			[63, 64, 67]
Dimethomorph	Cinnamic acid fungicide, used in agriculture (potatoes)				[66]
Endosulfan,	Organochlorine insecticide, banned by the Stockholm Convention (2011)	[61, 63, 67, 68, 70]		[61]	[63, 64, 66, 67, 69]
Endrin	Organochlorine pesticide, used in agriculture. Now banned in many countries	[61, 63]		[61]	[64]
Ethoxyquin	Quinoline-based antioxidant used as a food preservative (E324) and a pesticide				[66]
Etofenprox	Pyrethroid insecticide used in homes (pet and foggers)			[62]	
Fenarimol	Heterocyclic organochlorine fungicide, used on ornamentals, trees, horticulture (frequently used in Danish greenhouses in 2001)	[63, 67, 70]			[63, 67, 70]
Fenbuconazole	Triazole fungicide used in agriculture, fruit	[61]			
Fenhexamid	Fungicide, used on fruit, ornamentals, trees				[66]
Fenitrothion	Organophosphorus insecticide, agriculture, grain storage				[66]
Fenthion	Organothiophosphate insecticide, avicide, and acaricide, widely used in sugar cane, rice, field corn, beets, pome and stone fruit, citrus fruits, pistachio, cotton, olives, coffee, cocoa, vegetables, and vines				[71]
Fenvalerate	Pyrethroid pesticide, agriculture, home, garden	[61, 63]			[63]
Fludioxonil	Fungicide used on sowing-seed, vegetables, fruits, and ornamentals				[66]
Imazalil	Imidazole fungicide: sowing seed, potatoes, and fruits after harvesting				[66]
Lindane	Organochlorine insecticide, used to treat lice and scabies				[64]
Linuron	Phenylureum herbicide: horticulture				[66]
Methiocarb	Carbamate insecticide, still frequently used in Danish greenhouses in 2001	[63, 67, 68]			[63, 66, 67]
Methoxychlor	Organochlorine pesticide, used on crops, ornamentals, livestock, and pets against insects. Banned in the USA (2003) and European Union (2002)	[22, 61, 63, 67]		[61, 63]	[63, 64]
Metolachlor	Chloroacetanilide herbicide, grass and broadleaf weed control in agriculture	[63]			
O-Phenylphenol	Fungicide, generally applied after harvest; used for waxing citrus fruits. As a food additive, it has E number E231				[66]
Pentachlorophenol	Organochlorine pesticide: herbicide, insecticide, fungicide, antifouling paint, preservation of wood. Use now restricted			[61]	[64]
Permethrin	Pyrethroid insecticide, agriculture, home, garden	[63]		[62]	[69]
Pirimicarb	Carbamate insecticide: vegetable, cereal, and orchard crops	[68]			
Pirimiphos-methyl	Phosphorothioate insecticide: postharvest insecticide used on stored corn and sorghum grain and seed				[66]
Prochloraz	Imidazole-type foliar fungicide: gardening and agriculture, still frequently used in Danish greenhouses in 2001.	[72, 73]			[60, 66, 67]
Procymidone	Dicarboximide fungicide: preharvest spray or postharvest dip of lupins, grapes, stone fruit, strawberries				[66]
Propamocarb	Carbamate fungicide: horticulture, ornamentals, agriculture	[68]			
Pyrazoxyfen	Benzoylpyrazole herbicide			[63]	
Pyrimethanil	Fungicide: fruit,vineyards				[66]
Quinoxyfen	Fungicide: cereals, beets, strawberries, some berries				[66]
Tebuconazole	Triazole fungicide: agriculture. Assessed by the Swedish Chemicals Agency as potentially toxic;				[66]
Tetramethrin	Pyrethroid insecticide: Primarily pressurized aerosols in household, industrial, commercial, and institutional settings			[74]	
Tolclofos-methyl	Organophosphorous fungicide, still frequently used in Danish greenhouses in 2001	[63, 67, 68]			
Toxaphene	Organochlorine insecticide (banned in many countries)	[61, 68]			
Trans-nonachlor	Organochlorine insecticide: agriculture	[61]			[64]
tribenuron-methyl	Herbicide: grains; still frequently used in Danish greenhouses in 2001	[67]			
Triflumizole	Conazole fungicide: plant protection			[63]	
Vinclozolin	Dicarboximide fungicide, used on fruits and vegetables, still frequently used in Danish greenhouses in 2001. Use now strictly regulated	[61]			[63, 66, 67]

^a^These data are intended to give some indication, but are far from complete.

**Table 2:** Some epidemiological studies on breast cancer.

Exposure	Study population	Relative risk or odds ratio (95% confidence interval)	Main strengths	Main limitations	Reference
Ecological studies					

2,3,7,8-tetrachlorodibenzo-p-dioxin	Inhabitants of Seveso region	Rate ratio (heavily contaminated zone versus control zone): 2.57 (1.07–6.20)	Environmental exposure and time since exposure well documented,	Based on only five cases	[87]

Organochlorine pesticide methoxychlor	California Latinas commonly employed in agricultural operations	OR for highest quartile of county-level specific pesticide use: 118 (1.03–1.35)	Large number of cases	Poor evaluation of exposure. Incomplete correction for confounding	[88]
Organochlorine pesticide toxaphene		OR for highest quartile of county-level specific pesticide use: 116 (1.01–1.34)			

Case-control studies					

Polychlorinated biphenyls (20 congeners)	Mexican Women, hospital based	OR for serum concentration above detection limit: 1.09 (1.01–1.14)	Measurement of personal internal exposure. Adequate correction for confounders	Limited number of subjects	[50]
Polychlorinated biphenyl congeners 128, 138 and 170		OR for serum concentration above detection limit: 1.90 (1.25–2.88)			
Polychlorinated biphenyl congeners 153,180		OR for serum concentration above detection limit: 1.81 (1.08–3.04)			
Polychlorinated biphenyl congeners 8, 195, 206 and 209		OR for serum concentration above detection limit: 1.57 (1.20–2.07)			

Monoethyl phthalate (main diethyl phthalate metabolite)	Northern Mexico, population based	OR for highest versus lowest tertile of urinary concentration: 2.20 (1.33–3.63)	Measurement of personal internal exposure. 233 cases and 221 controls. Adequate correction for confounders	Metabolites of other phthalates were inversely associated	[89]

Total effective organohalogen xenoestrogen burden in adipose tissue	Hospital based, Southern Spain	OR for women with BMI below the median, highest quartile versus lowest of adipose tissue concentration: (28.6 kg/m^2^): 2.44; (1.03–5.78); *P*_trend_ = 0.03	Highly relevant parameter of personal internal exposure. Adequate correction for confounding.	Significant effect only in subgroup of leaner women.	[90]
Total effective organohalogen xenoestrogen burden in adipose tissue	Hospital based, Southern Spain	OR for postmenopausal women with BMI below the median (28.6 kg/m^2^), highest terile versus lowest of adipose tissue concentration: 5.6 (1.59–20.21)	Highly relevant parameter of personal internal exposure. Adequate correction for confounding	Significant effect only in subgroup of leaner women	[90]
Aldrin		OR for detectable adipose tissue concentration: 1.55 (1.00–2.40)			
Aldrin		OR among postmenopausal women for detectable adipose tissue concentration: 1.84 (1.06–3.18)		No effect observed for other organochlorine pesticides	
Lindane		OR among postmenopausal women for detectable adipose tissue concentration: 1.76 (1.04–2.98)			
Dioxin	Residents near municipal solid waste incinerator. Population based	OR for women aged ≥ 60 in region with high exposure: 0.31 (0.08–0.89)	High total number of cases (434) and controls (2170).	Exposure defined by area of residence; only 4 cases in highly exposed area. No effect on younger women. Limited correction for confounding.	[91]
Urinary cadmium levels	Population based in Wisconsin, USA	Highest quartile of creatinine-adjusted cadmium level (≥0.58 *μ*g/g) versus lowest quartile (<0.26 *μ*g/g) OR: 2.29 (1.3–4.2); *P*_trend_ = 0.01	Measurement of personal internal exposure. Adequate correction for confounding		[92]
Traffic emisions	Women residing in Erie and Niagara Counties, USA	Exposure at the time of menarche: OR for premenopausal breast cancer for highest quartile of exposure: 2.05 (0.92–4.54, *P*_trend_ = 0.03)	Adequate correction for confounding	Limited characterization of exposure. Limited statistical power	[93]
Traffic emmisions		Exposure at the time of a woman's first birth: OR for postmenopausal breast cancer for highest quartile of exposure: 2.57 (1.16–5.69, *P*_trend_ = 0.19)			
Exposure as motor vehicle mechanics	Multicentre case-control study on male breast cancer in 8 European countries, including 104 cases and 1901 controls	OR for ever employment: 2.1, (1.0–4.4).OR for employment ≥10 years: 5.9 (2.4–14.6)	Adequate correction for confounding	Limited characterization of exposure	[94]
Alkylphenolic compounds		OR for exposure above median: 3.8 (1.5 to 9.5)			

Cohort studies					

Dieldrin	Participants in the Copenhagen City Heart Study	OR for highest quartile of serum concentration 2.05 (1.17–3.57), *P*_trend_ = 0.01	Prospective design. Long follow-up. Adequate correction for confounding Individual internal exposure measured long before diagnosis.	Limited statistical power. No effects of other organochlorines	[95]
Solvents	Retrospective cohort study on 63,982 female workers in an electronics factory in Taiwan	SIR for women first employed prior to 1974: 1.38 (1.11–1.70)	Large number of subjects	Poor characterization of exposure; no correction for confounders	[96]
Volatile organic chemicals	274,596 women in active duty in the US army (1980–1996)	Incidence rate ratio for medium or high potential solvent exposure: 1.48 (1.01–2.07)	Large number of subjects	Poor characterization of exposure. Poor correction for confounders	[97]

Prospective nested case control study					

P,p^′^-DDT	Members of the Child Health and Development Cohort, Oakland, California	OR for highest tertile of serum concentration among women under age 14 in 1945: 5.4 (1.7–17.1)	Measurement of personal internal exposure. at highly relevant age. Adequate control for confounding	No effect for other DDT-related compounds	[98]

**Table 3:** Some epidemiological studies on prostate cancer.

Exposure	Study population	SMR, relative risk or odds ratio (95% confidence interval)	Main strengths	Main limitations	Reference
Ecological studies					

Arsenic	Members of the Mormon church from Millard County, Utah	SMR compared to the general population of Utah: 1.45 (1.07–1.91)	Quite good characterisation of exposure. Cohort population with restrictive life style	No clear dose-effect relationship; no correction for confounding	[105]
Arsenic	Residents of 314 of the 361 districts in Taiwan	Multivariate-adjusted regression coefficient for age-adjusted mortality per 100,000 person-years for every 0.1 ppm increase in arseniclevel of well water: 0.5 (SE: 0.2) *P* < 0.05	Quite good characterisation of exposure	Incomplete correction for confounding	[106]

Case-control studies					

Polychlorinated Biphenyl congener 153		OR for greater than median adipose tissue concentration: 3.15 (1.04–9.54)			
Trans-chlordane		OR for greater than median adipose tissue concentration: 3.49 (1.08–11.2)			
Polychlorinated Biphenyl congener 153	General population of Örebro County, Sweden	OR for prostate cancer with PSA > median, for greater than median adipose tissue concentration: 30.3 (3.24–284)	Highly relevant parameter of personal internal exposure. Adequate correction for confounding.	Small number of subjects	[107]
Trans-chlordane		OR for prostate cancer with PSA > median, for greater than median adipose tissue concentration: 11.0 (1.87–64.9)		Small number of subjects. The biological relevance of the arbitrary cut-off point of PSA is unclear	
Hexachlorobenzene		OR for prostate cancer with PSA > median for greater than median adipose tissue concentration: 9.84 (1.99–48.5),			

Cohort studies					

Polychlorinated Biphenyls	Electrical Capacitor Manufacturing Workers in New York and Massachusetts	Prostate cancer mortality increased with cumulative exposure to RR = 6.05 for the highest quartile (trend *P* value = 0.0001)	Quite elaborate characterisation of exposure	Limited correction for confounding	[108]

Beta-hexachlorocyclohexane		ORs for the 2^°^ and 3^°^ tertiles of detectable values were 1.46 (0.52–4.13) and 3.36 (1.24–9.10) (*P* for trend = 0.02)			
Trans-nonachlor	Participants in the U.S. National Health and Nutrition Examination Survey (1999-2000)	ORs for the 2^°^ and 3^°^ tertiles of detectable values were 5.84 (1.06–32.2) and 14.1 (2.55–77.9) (*P* for trend = 0.002)	Measurement of personal internal exposure. Adequate correction for confounding	Self-reported cancer diagnosis. Only 65 cases	[109]
Dieldrin		ORs for the 2^°^ and 3^°^ tertiles of detectable values were 1.06 (0.30–3.73) and 2.74 (1.01–7.49) (*P* for trend = 0.04)			

Prospective cohort studies					

Application of pesticides	Participants in the Agricultural Health Study in Iowa and North Carolina	OR: 1.14 (1.05, 1.24) compared to male populations of Iowa and North Carolina	Participants: 55,332 male pesticide applicators Elaborate characterization of external exposure. Adequate correction for confounding	Short followup period	[110]

Meta-analysis					

Application of pesticides	22 studies (1986–2003)	RR: 1.24 (1.06–1.45) RR's higher in North America than in Europe	RR's homogenous in same region No bias	Limited info on specific chemicals	[111]
Manufacturing of pesticides	18 studies (1984–2004)	RR: 1.28 (1.05–1.58). Consistent increase for all chemical classes, significant for phenoxy herbicides	No publication bias	Limited info on specific chemicals	[112]

**Table 4:** Some epidemiological studies on non-Hodgkin lymphoma.

Exposure	Study population	Relative risk or odds ratio (95% confidence interval)	Main strengths	Main limitations	Reference
Ecological studies					

Residing in vicinity of municipal solid waste incinerators with high dioxin emission levels	Population of four French administrative department (Isère, Bas-Rhin, Haut-Rhin, Tarn)	RR for living in highly exposed area compared to living in slightly exposed area: .120 (1.002–1.251)	High number of cases. Acceptable characterization of environmental exposure	Limited correction for confounding (only on geographical block level)	[141]

Case control studies					

*β*-hexachlorocyclohexane		OR per 10 ng/g serum lipid: 1.05 (1.00–1.12)			
p,p^′^ dichloro-diphenyl-trichloroethane (DDT)		OR per 10 ng/g serum lipid: OR = 1.20 (1.01–1.45)			
Dioxins	Neighbors of a French municipal solid waste incinerator	OR per pg WHO1998-TEQ/g lipid.: 1.12 (1.03–1.26) *P* < 0.01	Measurement of personal internal exposure. Adequate correction for confounders	Only 34 cases and 34 controls	[142]
Furans		OR per pg WHO1998-TEQ/g lipid. 1.16 (1.03–1.35) *P* = 0.01			
Dioxin-like PCBs		OR per pg WHO1998-TEQ/g lipid.: 1.04 (1.00–1.07) *P* = 0.02			
Non-dioxin-like PCBs		OR per 10 ng/g serum lipid: 1.02c (1.01–1.05) *P* = 0.01			

**Table 5:** Some epidemiological studies on diabetes.

Exposure	Study population	Relative risk or odds ratio (95% confidence interval)^a^	Main strengths	Main limitations	Reference
Ecological studies					

Dioxin TCDD	Mortality from diabetes in the cohort involved in the Seveso accident	OR for death from diabetes was increased in all contaminate area's. OR in zone with substantial contamination: 1.9 (1.1–3.2)	Environmental exposure and time since exposure well documented	Only 13 cases	[191]

Cross-sectional studies					

Seventeen 2,3,7,8-polychlorinated dibenzodioxins/dibenzofurans		OR for top decile of adjusted serum concentrations of dioxins: 5.1 (1.18–21.7)			
Coplanar polychlorinated biphenyls 77, 81, 126 and 169	Population-based study in Belgium	OR for top decile of adjusted serum concentrations of dioxin-like PCBs 13.3 (3.31–53.2)	Precise measurement of personal internal exposure. Adequate control of confounding	Only 9 cases	[192]
Marker polychlorinated biphenyls		OR for top decile of adjusted serum concentrations of marker PCBs: 7.6 (1.58–36.3)			

Polychlorinated biphenyls	Cross-sectional study on 2,245 Pregnant women from the Boston (USA) area	Compared to <2.5 *μ*g/mL serum, OR for 2.5–<3.75 *μ*g/mL: 2.9 (1.1–7.3); for 3.75–<5.0 *μ*g/mL: 4.4 (1.6–12.5) for >5.0 *μ*g/mL: 5.1 (1.9–13.8) (*P*_trend_ = 0.004)	Precise measurement of personal internal exposure. Adequate control of confounding	Only 44 cases	[193]

2,3,7,8-Tetrachloro- dibenzo-p-dioxin (TCDD)	Subjects in good health residing within 25 miles of Vertac/Hercules Superfund site in Jacksonville, Arkansas	OR for fasting insulin level >4.5 *μ*IU/mL for top decile TCDD serum concentration (>15 ppt) : 8.5 (1.49–49.4)	Precise measurement of personal internal exposure. Adequate control of confounding	Limited number of subjects	[194]

2,2′,4,4′,5,5′-hexachlorobiphenyl (CB-153)	196 Swedish fishermen (median age 60 years) and their wives (median age 64 years, *n* = 184),	OR for an increase of 100 ng/g serum lipid: 1.16 (1.03, 1.32) *P* = 0.03	Precise measurement of personal internal exposure. Adequate control of confounding	Only 22 cases of diabetes	[195]
1,1-dichloro- 2,2-bis(p-chlorophenyl)-ethylene (p,p′-DDE)		OR for an increase of 100 ng/g lipid: 1.05 (1.01, 1.09), *P* = 0.006)			

Polychlorinated biphenyl 153		OR for highest decile of serum concentration compared to not detectable: 6.8 (3.0–15.5) *P*_trend_ < 0.001			
1,2,3,4,6,7,8-heptachlorodibenzo-p-dioxin		OR for highest decile of serum concentration compared to not detectable: 2.7 (1.3–5.5) *P*_trend_ = 0.007		No differentiation between type I and type II. Few cases in referent exposure category	
1,2,3,4,6,7,8,9-octachlorodibenzo-p-dioxin		OR for highest decile of serum concentration compared to not detectable: 2.1 (0.9–5.2) *P*_trend_ = 0.094			
Oxychlordane		OR for highest decile of serum concentration compared to not detectable: 6.5 (2.0–21.4) *P*_trend_ < 0.001			
DDE	2,016 adult participants in the National Health and Nutrition Examination Survey 1999–2002	OR for highest decile of serum concentration compared to lowest quartile: 4.3 (1.8–10.2) *P*_trend_ = 0.001	Precise measurement of personal internal exposure. Adequate control of confounding	No differentiation between type I and type II	[196]
Trans-nonachlor		OR for highest decile of serum concentration compared to not detectable: 11.8 (4.4–31.3) *P*_trend_ < 0.001		No differentiation between type I and type II. Few cases in referent exposure category.	
Cumulative measure^b^ of six organochlorines		OR for highest decile of serum concentration compared to lowest quartile: 37.7 (7.8–182) *P*_trend_ < 0.001		No differentiation between type I and type II	
Cumulative measure^b^ of six organochlorines		OR for highest decile of serum concentration compared to second quartile: 2.7 (1.5–4.8) *P*_trend_ < 0.001			

Cumulative measure^c^ of 1,2,3,6,7,8-hexachlorodibenzo-p-dioxin, 1,2,3,4,6,7,8-heptachlorodibenzo-p-dioxin, and 1,2,3,4,6,7,8,9-octachloro-dibenzo-p-dioxin		OR for highest quartile of serum concentration compared to lowest quartile: 1.8 (1.0–3.3). *P*_trend_ < 0.001			
Cumulative measure^c^ of 2,3,4,7,8-pentachlorodibenzofuran, 1,2,3,4,7,8-hexachlorodibenzofuran and 1,2,3,4,6,7,8-heptachloro dibenzofuran		OR for highest quartile of serum concentration compared to lowest quartile: 3.7 (2.0–7.1)			
Cumulative measure^c^ of Dioxin-like PCBs (PCBs 74, 118, 126, 156)		OR for highest quartile of serum concentration compared to lowest quartile: 24.3 (7.0–84.5)			
Cumulative measure^c^ of nondioxin like PCBs (PCBs 138, 153, 170, 180, 187)	1,721 participants in the National Health and Nutrition Examination Survey 1999–2002, aged ≥ 20 years 1	OR for highest quartile of serum concentration compared to lowest quartile: 6.2 (2.8–13.6)	Precise measurement of personal internal exposure. Adequate control of confounding	Rather low number of cases in lowest quartile of exposure	[197]
Cumulative measure^c^ of organochlorine pesticides (p,p_-Dichlorodiphenyltrichloroethane, oxychlordane, trans-nonachlor, Beta-hexachlorocyclohexane)		OR for highest quartile of serum concentration compared to lowest quartile: 13.9 (5.0–38.7)			
Trans-nonachlor		OR for highest quartile of serum concentration compared to not detectable: 8.0 (2.6–24.8)		Few cases in referent exposure category	
Beta-hexachlorocyclohexane		OR for highest quartile of serum concentration compared to not detectable: 7.0 (2.7–18.1)			

Cumulative measure^c^ for Organochlorine pesticides (Oxychlordane, Trans-nonachlor, p,p-dichlorodiphenyltrichloroethane and -Hexachlorocyclohexane)		OR for HOMA-IR insulineresistance values >90th percentile for highest quartile of serum concentration compared to lowest quartile: 7.5 (2.3–23.9) *P*_trend_ < 0.01		Only 12 subjects with high values for insuline resistance in lowest quartile of exposure	
Oxychlordane		OR for HOMA-IR insuline resistance values >90th percentile for highest quartile of serum concentration compared to “nondetectable”: 8.7 (2.3–33.3) *P*_trend_ < 0.01		Only 8 subjects with high values for insuline resistance in referent exposure category	
Trans-nonachlor	749 nondiabetic participants in the National Health and Nutrition Examination Survey 1999–2002 aged ≥ 20 years.	OR for HOMA-IR insuline resistance values >90th percentile for highest quartile of serum concentration compared to “nondetectable”: 5.4 (1.3–23.1) *P*_trend_ = 0.02	Precise measurement of personal internal exposure. Adequate control of confounding	Only 4 subjects with high values for insuline resistance in referent exposure category	[198]
p,p-dichlorodiphenyltrichloroethane		OR for HOMA-IR insuline resistance values >90th percentile for highest quartile of serum concentration compared to lowest quartile: 1.4 (0.5–3.7) *P*_trend_ = 0.33		Only 15 subjects with high values for insuline resistance in lowest quartile of exposure	
Hexachlorocyclohexane		OR for HOMA-IR insuline resistance values >90th percentile for highest quartile of serum concentration compared to “nondetectable” 1.7 (0.6–5.1) *P*_trend_ = 0.56		Only 15 subjects with high values for insuline resistance in referent exposure category	

Polybromobiphenyl-153	1,367 adults participating in the National Health and Nutrition Examination Survey 2003-2004	OR for highest quartile of serum concentration compared to “nondetectable”: 1.9 (0.9–4.0). *P*_trend_ = 0.01	Precise measurement of personal internal exposure. Adequate control of confounding	Only 11 subjects with diabetes in referent exposure category	[199]

Sum of di(2-ethylhexyl) phthalate (DEHP) metabolites	221 Healthy Mexican women serving as controls in a case-control study for breast cancer	Odds ratios for log transformed urinary phthalates metabolites concentration and self-reported diabetes: 1.64 (0.98–2.73). *P* = 0.060	Precise measurement of personal internal exposure. Adequate control of confounding	Only 39 diabetes cases	[200]

CDDs + PCDFs (pg TEQ/g lipid)	1374 subjects, not occupationally exposed to dioxins, aged 15–73 years, living throughout Japan (2002–2006)	OR for highest quartile of serum concentration compared to 1st plus 2nd quartile: 2.21 (1.02–5.04)	Precise measurement of personal internal exposure. Adequate control of confounding	No distinction between types of diabetes. Reference category includes 1st and 2nd quartiles	[201]
Dioxin-like PCBs (pg TEQ/g lipid)		OR for highest quartile of serum concentration compared to 1st plus 2nd quartile: 6.82 (2.59–20.1)			

Prospective studies					

Oxyclordane	Nested case-control study within the Coronary Artery Risk Development in Young Adults (CARDIA) cohort	OR for highest quartile of serum concentration compared to lowest quartile: 2.6 (1.0–7.0)	Precise measurement of personal internal exposure. Adjusted for age, sex, race, BMI	Correlation weakened by Additional correction for blood lipids	[202]
Trans-nonachlor		OR for 2nd quartile of serum concentration compared to lowest quartile: 4.8 (1.7–13.7)		Only 7 cases in lowest quartile (reference)	

PCB153	Nested case-control study within the Coronary Artery Risk Development in Young Adults (CARDIA) cohort.	OR for 2nd quartile of serum concentration compared to lowest quartile: 2.4 (1.0–6.1)	Precise measurement of personal internal exposure. Adjusted for age, sex, race, BMI	Correlation weakened by additional correction for blood lipids	[202]
PCB 180		OR for 2nd quartile of serum concentration compared to lowest quartile: 3.4 (1.3–9.0)			
PCB170		OR for 2nd quartile of serum concentration compared to lowest quartile: 2.7 (1.0–7.1)		Correlation weakened by additional correction for blood lipids	
PBB-153		OR for 3rd quartile of serum concentration compared to lowest quartile		Correlation weakened by additional correction for blood lipids	

^a^Odds ratio's are for diabetes, except when insulin resistance is mentioned.

^b^To calculate the cumulative measure, the category number of each POP (0 assigned to the nondetectable category, and 1 through 5 assigned to successively increasing categories) was added to make the sum of POP levels (SUMPOPs), producing a value of 0–30, which was itself categorized at its 25th, 50th, 75th, and 90th percentiles, making five groups.

^c^To calculate the cumulative measure of three PCDDs, the ranks of the three PCDDs were summed, then divided into quartiles. Cumulative measures were calculated in the same way subclasses similarly for the three PCDFs, the four dioxin-like PCBs, the five nondioxin-like PCBs, and the four organochlorine pesticides.

**Table 6:** Some cross-sectional epidemiological studies on metabolic syndrome.

Exposure	Study population	Relative risk or odds ratio (95% confidence interval)	Main strengths	Main limitations	Reference
Polybromobiphenyl-153	367 Adults participating in NHANES 2003-2004	OR for highest quartile of serum concentration compared to “non detectable”: 3.1 (1.4–6.9) *P*_trend_ < 0.01	Precise measurement of personal internal exposure. Adequate control of confounding. 237 cases	Only 18 cases in referent category	[199]
PBDE-153		OR for second quartile^a^ of serum concentration compared to “non detectable”: 2.5 (1.1–5.6). *P*_trend_ = 0.02^a^		4 other PBDEs did not show a significant association	

Cumulative measure^b^ of PCDDs		OR for highest quartile of serum concentration compared to lowest quartile: 1.3 (0.7–2.5) *P*_trend_ = 0.35			
Cumulative measure^b^ of PCDFs		OR for highest quartile of serum concentration compared to lowest quartile: 1.6 (0.9–2.8). *P*_trend_ = 0.11			
Cumulative measure^b^ of Dioxin-like PCBs	721 Nondiabetic participants in the National Health and Nutrition Examination Survey 1999–2002 aged ≥ 20 years	OR for highest quartile of serum concentration compared to lowest quartile: 2.1 (1.0–4.3). *P*_trend_ = 0.01	Precise measurement of personal internal exposure. Adequate control of confounding^c^. 175 cases	Positive pairwise correlations between the different classes of pops	[224]
Cumulative measure^b^ of non-dioxin-like PCBs		OR for third quartile^a^ of serum concentration compared to lowest quartile: 1.8 (1.0–3.4) *P*_trend_ < 0.01^a^			
Cumulative measure^b^ of organoclorine pesticides		OR for highest quartile of serum concentration compared to lowest quartile: 5.3 (2.5–11.3). *P*_trend_ < 0.01			

PCDDs	1,374 subjects not occupationally exposed to these pollutants, living throughout Japan during 2002–2006	OR for highest quartile of serum concentration compared to lowest quartile: 3.2 (1.6–6.7). *P*_trend_ < 0.01	Precise measurement of personal internal exposure Adequate control of confounding	Use of BMI instead of waist circumference and of glycated haemoglobin instead of serum glucose	[225]
PCDfs		OR for highest quartile of serum concentration compared to lowest quartile: 4.4 (2.0–11). *P*_trend_ = 0.04			
Dioxin-like PCBs		OR for highest quartile of serum concentration compared to lowest quartile: 4.8 (2.2–11). *P*_trend_ < 0.01			

^a^An inverted U-shaped association was observed, and the *P* value for quadratic term is shown.

^b^To yield a cumulative measure of pops belonging to a specific class, such as PCDDs or organochlorine pesticides, the ranks of the individual pops were summed. The summary values were categorised by cut-off points of 25th, 50th, and 75th percentile values.

^c^Adjusted for age, sex, race, poverty income ratio, cigarette smoking, serum cotinine, alcohol consumption, and exercise.

## Acronyms and Abbreviations

4-OHT: 4-hydroxytamoxifen
6-OH-BDE-47: Hydroxylated metabolite of 2,2′, 4,4′-tetra-bromodiphenylether(BDE-47)
AhR: Aryl hydrocarbon receptor
AP-1: Activator protein 1 (gene regulatory protein)
AR: Androgen receptor
Arnt: Aryl hydrocarbon receptor nuclear translocator (gene regulatory protein collaborating with the Ah receptor)
BMI: Body Mass Index
BPA: Bisphenol A
cAMP: Cyclic adenosine monophosphate
CAR: Constitutive androstane receptor
CDC: United States Centre for Disease Control and Prevention
CI: Confidence Interval
DDD: (1,1-dichloro-2,2-bis(p-chloro*­*phenyl)ethane) (DDT metabolite)
DDE: Dichlorodiphenyldichloroethylene
DDT: 1,1,1-trichloro-2,2-bis(p-chlorophenyl)ethane) (DDT metabolite)
DEHP: Bis(2-ethylhexyl) phthalate
DES: Diethylstilbestrol
E2: 17beta-estradiol
E3: Estriol
EDC(s): Endocrine-disrupting compound(s)
EE2: 17*α*-ethynylestradiol
eNOS: Endothelial NO synthase (enzyme producing nitric oxide)
EP: Ethylphenol
EPA: Environmental Protectio Agency
ER: Estrogen receptor
ERE(s): Estrogen responsive element(s)
ERK: Mitogen-activated protein kinase (signal transducing)
ERR(s): Estrogen-related receptor(s)
ER*α*: Estrogen receptor alfa
ER*β*: Estrogen receptor beta
E-screen: Test for detecting oestrogenic activity
F1 generation: First generation after treated animals
F2, F3, F4: Generations following the F1generation
FSH: Follicle stimulating hormone
GC/MS: Gas chromatography–mass spectrometry
GnRH: Gonadotropin-releasing hormone
GPR30: G-protein-coupled receptor 30
hAR: Human androgen receptor
HDL: High density lipoprotein
Hepta-BDE: Heptabrominated diphenyl ether
HOMA-IR: Homeostasis model assessment-insulin resistance (measure of insulin resistance)
HSD(s): hydroxysteroid dehydrogenase(s)
IRR: Incidence rate ratio
LH: Luteinizing hormone
MAP: Mitogen-activated protein (gene regulatory protein)
MBP: Mono-n-butyl phthalate
MBzP: Monobenzyl phthalate
MEHHP: Mono-2-ethyl-5-hydroxyhexyl phthalate
MEOHP: Mono-2-ethyl-5-oxohexyl phthalate
mRNA: Messenger Ribonucleic Acid
MVLN cells: MCF -7 human breast carcinoma cells with an estrogen receptor controlled luciferase reporter gene
NF*κ*B: Nuclear factor kappa-light-chain-enhancer of activated B cells (gene regulatory protein)
NHANES: National Health and Nutrition Examination Survey
NIESH: National Institute of Environmental Health Sciences
NO: Nitric oxide
NOS: NO synthase (enzyme producing nitric oxide)
NP: Nonylphenol
OC pesticides: Organochlorine pesticides
OP: Octylphenol
OR(s): Odds ratio(s)
P300: Transcriptional coactivator (gene regulatory protein)
PBB-153: Polybrominated biphenyl-153
PBDE(s): Polybrominated diphenyl ether(s)
PCB 118: 2,3′, 4,4′, 5-Pentachlorobiphenyl (a mono-ortho Polychlorinated biphenyl)
PCB(s): Polychlorinated biphenyl(s)
PCDD(s): Polychlorinated dibenzo-p-dioxin(s)
PCDF(s): Polychlorinated dibenzofuran(s)
PCOS: Polycystic ovary syndrome
pERK: Phosphorylated (activated) ERK
PFOA: Perfluorooctanoic acid
PI3K: Phosphatidylinositol 3-kinase (signal transducing enzyme)
PKA: Protein kinase A (signal transducing enzyme)
PKB: Protein kinase B (also designated AKT) (signal transducing enzyme)
PKC: Protein kinase C (signal transducing enzyme)
POF: Premature ovarian failure
POP(s): Persistent organic pollutant(s)
PP: Propylphenol
p,p′-DDE: 1, 1′-dichloro-2, 2′-bis(p-chlorophenyl) ethylene
p,p′-DDT: 1,1,1-trichloro-2,2-bis(pchlorophenyl) ethane
PRL: Prolactin (hormone)
PXR: Pregnane X receptor
RAS: Signal transducing protein, coded for by a protooncogene, belonging to the Ras superfamily of monomeric Guanosine Triphosphate Phosphatases
RR: Relative risk
SMR: Standardized mortality ratio
Sp1: Specificity protein 1 (gene regulatory protein)
Src: Signal transducing protein tyrosine kinase belonging to the Src family of kinases (coded for by a protooncogene)
Src/Ras/Erk: Important intracellular signalling pathway
STAT(s): Signal transducer(s) and activator(s) of transcription (gene regulatory protein(s))
T3: 3,3,5-triiodo-L-thyronine
T4: Tetraiodo-L-thyronine
TBT: Tributyltin
TH: Thyroid hormone
TPT: Triphenyltin
TRH: Thyrotropin releasing hormone
TSH: Thyroid stimulating hormone
TTR: Transthyretin (blood transport protein for thyroxin)
UV: Ultraviolet light
WC: Waist circumference
YES: Yeast estrogen screen
*β*-HCH: *β*-hexachlorobenzene.
